# Observations on Obstetric Auscultation

**Published:** 1834-01-01

**Authors:** 


I. OBSERVATIONS ON OBSTETRIC AUSCULTATION. By *Evory Kennedy*, M.D. Dublin, pp. 288.

II. SIGNS OF PREGNANCY AND DELIVERY. By *W. F. Montgomery*.

[From Cyclopæd. of Pract. Med. p. 45.]

A MEDICAL man cannot possibly be long, or extensively engaged in the actual practice of his profession, wherever be the field of his labours, or the department of the science that he follows, without meeting with numerous cases, which are rendered doubtful and perplexing, by the difficulty of accurately determining, whether a woman be pregnant or not: the physician knows well that he ought to modify, or even alter his treatment of other existing affections, if the womb be at the time engaged in its special functions of forming and developing the embryotic germ; he knows well that the symptoms of every disease are often curiously blended with, and masked and rendered obscure by the co-existence of such a state; that the very state itself gives rise to a host of Proteian maladies, which defy alike nosological arrangement, and therapeutic relief; that the mind, as well as the body suffers singular changes; the mild and amiable becoming irascible, morose and fretful; the contented and happy forgetting their former cheerfulness, in capricious repining, and whimsical extravagances; and in short that his moral and corporeal treatment must be suited to the particular state of the system at the time.

Equally necessary is it to the surgeon to attend to the influence of pregnancy on those diseases, which are said to appertain to his sphere; he would be unwilling for example to perform any serious operation, which did not require to be done immediately, or he would at least be prepared for the probable occurrence of miscarriage, and regulate his conduct accordingly; he would remember that some maladies are inevitably aggravated by the presence of a fœtus in utero, and thus avoid disappointment to himself, as well as vexation, if not positively hurtful interference to his patient—and that others are temporarily arrested in their progress, or even altogether dissipated during pregnancy. But if this knowledge be of so much consequence to the general physician and surgeon, how doubly requisite is it for him who devotes himself specially to obstetrical practice. The public expect, as a matter of course, that an accoucheur be on all occasions able and ready to pronounce upon the existence or absence of impregnation, even when the woman herself is uncertain about it.

Unaware of the difficulties which sometimes surround the question, they suppose that a doctor, and especially if he be a married man ! should and ought to know at once, whether the fond hopes of the mother be rational or not; and they are apt, on receiving an equivocal reply, to attribute it to ignorance, inexperience, greedy self-interest, or some other equally discreditable motive. Now, although the "mens sibi conscia recti," regards as naught the insinuations of angry disappointment, or the abuse of malicious, although self-condemning shame, it cannot but be the desire of every honest and enlightened practitioner, that he was always enabled to decide at once and definitively on the interesting subject of existent pregnancy. The tenderest feelings of which the human breast is susceptible, would thank him for the information. The guilty consequences of concealment might often be prevented; the ridicule and sarcasm of the scurrilous world might be warded off; virtue might be protected from accusation, and crime be brought to proper punishment. We doubt that there is a situation in which a medical man can be placed professionally, so full of weighty consequences, and of anxious interest, as that of an accoucheur, in some cases, when called upon for his opinion as to the existence of pregnancy; the character, and all that is dear to a chaste woman may be foully blackened and ruined in a moment; property may be alienated from the path of justice to that of iniquity and vice; and even life itself may be, as we know that it has been more than once, sacrificed on the shrine of professional ignorance.

If then such fearful consequences may flow from medical evidence, how important is the duty of possessing an intimate acquaintance with every possible occurrence which can change or modify our opinions; and, unfortunately, on no theme of medical science is there more ambiguity and greater discrepancy of sentiments, than on the diagnosis of the pregnant state. True it is, that, even with the willing and rational assistance of the patient herself, the signs are sometimes so obscure and perplexingly variable, as to defy all confident assertion, and we are fairly obliged to confess that time alone can solve the problem; little wonder it is then, that the difficulties are ten-fold increased, when we have to combat with wilful deceit and false assertion on the one hand, or with the self-created imaginings of a longing fancy on the other. We must exercise all our tact and address in drawing out one particle of truth from the heap of lies or of nonsense which may be crammed into our ears; and nothing more strikingly exhibits the lynx-eyed sagacity of the skilful physician, and the rash forwardness of the impudent pretender, than the different conduct of the two upon such an occasion. The ever memorably-absurd case of Joanna Southcote, to which we shall allude more particularly in the sequel, is an apt illustration of these remarks. We now proceed to examine some of the most important of those evidences or signs of pregnancy, by which medical men are accustomed to be guided, in determining the existence of that state.

For the convenience of arrangement, as well as for the practical utility of an easy remembrancer, we shall briefly consider them in a four-fold point of view. The first embrace all those for the knowledge of which we are indebted solely to the patient herself, or to her immediate attendants, and which, for want of a better term, we shall call "oral," in contradistinction to the other three, which are ascertained by the physician himself, either with his eyes, his hands, or his ears, and may therefore aptly enough be termed, the visible, the tangible, and the audible signs of pregnancy. Without alluding to the uncertain and only occasional feelings which some women experience at the moment of conception, viz. those of intense, and almost maddening pleasurable excitement, and of pain, or something very like it, darting through their pelvis to the back, quickly followed by a state of drowsy exhaustion, we may mention that the female system very generally indicates soon, the curious change which has taken place, in one of its most important functions. There is a fretfulness or feverish irritability both of mind and body; the pulse becomes quickened, pains are felt in the loins and through the stomach, the head aches, and the organs of sense are sometimes unusually sensitive; the patient is easily agitated and alarmed; the bowels are either confined, or have a tendency to be relaxed, and not unfrequently there is considerable annoyance in passing water.

In the course of one, two, or three weeks, generally, the stomach begins to announce its sympathetic disturbance, so well known under the name of "morning sickness," and at this period sometimes, although it is usually later, the mammæ become swollen, painful, and tingling.

Now all these signs or symptoms of pregnancy may occur before the time at which the female should otherwise menstruate, and she, therefore, looks forward to the absence or return of this discharge as a confirmation, or not, of her suspicions. The vulgar, indeed, have always regarded the stoppage of the monthly courses, in a woman who was enjoying sexual connexion, as one of the surest, or at least of the most probable, indications of conception; and the opinion which is founded upon general observation is, doubtless, quite correct in the main; but if you tell a woman that it is quite possible for her to be in the family-way, and yet continue to menstruate, she will scarcely credit you, and little wonder is it, when we remember that some of the most experienced accoucheurs of modern times have distinctly stated—"that they never met with a single instance of any female continuing to menstruate when she was pregnant." Such are the words of the eminent Denman, no mean authority in midwifery. Yet read the conflicting assertion of Dr. Blundel.

"We must not conclude that a woman is not pregnant merely because she menstruates; for although doubts may be raised respecting the continuance of the catamenia during the whole term of gestation, yet I have repeatedly met with cases of pregnancy, in which the catamenia have continued to flow during the first two or three months; indeed this, notwithstanding Dr. Denman's assertion to the contrary, may, I think, be looked upon as by no means very uncommon."

Mauriceau tells us of a horrible case, which occurred in Paris in the year 1666. A woman was executed, although she swore that she was several months gone with child; the subject was referred by the judge to some persons, who were appointed to visit her. They reported that she was not pregnant—"because she had her monthly courses." On dissection, a four-months' fœtus was found in utero!!

From the almost unanimous opinions of accoucheurs of the present day, we are, therefore, bound to admit the possibility of the catamenia flowing for one, or for several successive periods, after impregnation; and our own experience confirms its accuracy. It has been said by some, that such discharges are not truly menstrual, but rather sanguineous, proceeding from the rupture of small vessels about the neck of the womb, and different authors have proposed means to distinguish the one sort from the other.

We are told of one eminent accoucheur, who placed so much dependance upon his knowledge of the sensible properties of the genuine catamenia, that he was in the habit of having towels sent to him from considerable distances, in order that he might distinguish the nature of the discharge from the stains. It has often indeed appeared to us rather singular, that women themselves should seldom or never be aware that there is any difference between the stains left by the menstrual flow, and those left by blood. We have often inquired of them, but never met with one who seemed to know that they were different; and the very custom of females resorting to the expedient of staining their linen with blood, for the purpose of deception (a deception which very often succeeds with their own sex), is another proof of it. As the menstrual fluid contains little or no fibrine, it does not properly coagulate, and, consequently, does not stiffen linen as blood does. Capuron says—"il faut exiger, alors, que les parties soient lavées avec l'eau tiede;—si le sang ne reparait pas, le cas est suspect." But we have heard of tricks of science to baffle this test; one of these is, to let a stream of blood flow into a cup of boiling water, and then to use this fluid as the dye—and another, which, by the bye, we accidentally discovered, when making some experiments on the action of different chemical agents on fresh-drawn blood, consists in dropping strong liquor ammoniæ into it. The following short notice we marked down at the time:—"The ammonia immediately caused the blood to assume a much darker colour throughout; only at the edges and on the sides of the cup, when it was inclined, it exhibited a pale redness; it also became thinner, and might be well compared to what is called 'sanious gore.' Six hours afterwards, the blood presented much the same appearance—it shewed no signs of separation into serum and clot, but remained quite fluid, dark-coloured, and tinging the inside of the cup with a pale reddish layer. When linen was stained with it, the spots resembled a good deal those from the menstrual discharge."

Although, however, we may be assisted somewhat in distinguishing the two sorts of stains, by attending to these marks we have noticed, it is right ever to keep in mind, that it is never safe to trust to them alone, and for this good reason, viz. that not unfrequently are the two discharges blended together in ordinary health, some of the small vessels giving way at the very time that the secretion is going on. No less unsatisfactory is the absence of the catamenia, as an indication of pregnancy, in another point of view; it is not, as is too well known, a symptom of this state exclusively, for during nursing, in chlorosis, in general and ovarian dropsy, in many chronic diseases, especially of the womb itself, the secretion is stopped ; and what adds much to our embarrassment is, that the very stoppage, or at least the state of the system with which it is connected, is very often attended with the other signs of impregnation—the tumid belly, the haggard looks, the morning sickness, and the painful breasts; add to these sometimes the counterfeit feelings of quickening, produced by the rolling about of wind in the bowels, or a convulsive twitching of the abdominal muscles, or by the pulsations of the aorta, &c. This last-mentioned sign, we mean that of quickening, is, indeed, an important one to be rightly understood, not only as to the varying characters of its actual occurrence, but also in regard to those phenomena which may simulate and be mistaken for it. It has acquired a very improper and a very dangerous distinction, from the legal consequences affixed to its having truly happened, or believed to have happened.

The terms "quick with child," "to be pregnant of a quick child," and such like, ought to be erased from the vocabulary of the law, as we shall explain more at large at a subsequent part of this paper ; for they imply a most ignorant presumption, and are derived from the vulgar prejudices, that the child in utero does not begin to live until the mother has felt the quickening motion. With regard to this symptom, no medical man in the present day will attach too much importance to it, when disjoined from, and unattended with, the other signs. The forcible and pithy observation of Dr. Conquest, in his evidence upon the Claims to the Gardiner Peerage is quite true :—"Many old women, who are determined to have children when they marry late in life, and many single women who wish not to have children, are very apt to be deceived." We have read of a case, where not only the lady vowed that she had felt the motions of the child within her, but also her husband, a medical man, too, was equally assured that he had recognized the kicking and thumping of the little one through the abdominal parietes; and yet the pregnancy was one of some watery cysts in the uterus. The knowledge of such facts ought never to be lost sight of ; and their importance is enhanced, by the occasional completion of genuine and perfect pregnancy, without this symptom having been even once recognized by the patient.

### Visible Signs of Pregnancy.

The most important of these are derived from the inspection of the mammæ, of the abdomen, and of the urine.

The mere enlargement and painful state of the mammary glands are not much to be depended upon, as the same changes take place in a multitude of other affections, especially in whatever involve the generative organs ; we shall, therefore, limit our remarks, at present, to the subject of the dark circle, or areola, which is formed round the nipples of pregnant women. Here, as upon so many other occasions, we shall be obliged to admit the truth of the old taunting proverb—"doctors differ." Drs. Smellie and Wm. Hunter regarded the formation of this dark ring, as proof positive and conclusive of pregnancy ; and the latter eminent physician is "said to have placed such confidence in it, that he on one occasion pronounced a female subject, in the dissecting-room, pregnant upon this single sign, although the hymen was perfect; and his opinion, on dissection, proved correct.* On the other hand, Dr. Denman, with all his practical opportunities and acute powers of discrimination, has arrived at a very different estimate of its value."

Dr. Montgomery supposes that much of the discrepancy of opinion upon this subject has arisen from inaccuracy in observing and describing the essential characters of the true areola; too much attention has been paid to the mere change of colour, and too little to the other accompanying phenomena. He, therefore, adduces the description left by Rœderer, as the only proper test by which to judge of the value of this sign. The words are—

"Menstruorum suppressionem mammarum tumor insequitur; quocirca mammæ crescunt, replentur, dolent interdum, indurescunt: venæ earum cœruleo colore conspicuæ redduntur, *crassescit papilla, inflata videtur, color ejusdem fit obscurior, simili colore distinguitur discus ambiens qui in latitudinem majorem expanditur, parvisque eminentiis, quasi totidem papillulis, tegitur.*"† 9.

All these individual appearances ought to be observed, and, in addition to them, a soft and moist state of the integument, and occasionally a slight oozing from the little glandular follicles, sufficient to damp and colour the woman's inner dress. We are not to expect to find these changes before the end of the second month; at the end of the fourth, they are generally perfected. Dr. M. is inclined to place very considerable confidence in the indications to be drawn from the state of the nipple and of its areola. When all the appearances above described are coexistent, he says, "they are marks of great value, and in experience have never deceived us; and we certainly never saw any other condition of the part produced by disease, which could possibly be mistaken for them."

The secretion of milk, or of a milky fluid, from the breasts, is a sign which we cannot well rely upon; for not only can some women, who have had children, work a little milk out at all times, but old women and young maids, and even our own rougher sex, have occasionally acted the part of a wet-nurse—*see* Humboldt's Personal Narrative, and the Bishop of Cork's paper in Phil. Trans. for 1741.

The other ocular signs of pregnancy, such as the size of the abdomen, the protrusion of the umbilicus, the swelling, œdema, and varicose state of the lower extremities, and the peculiar sharpening of the features, although worthy of notice, are of very inferior value if taken by themselves. The chemical test lately proposed by M. Nanche, has been found by Mr. Kane, of Dublin, to be quite inapplicable. M. Nanche had stated—

"That pregnancy may always be detected by 'allowing the urine of pregnant women or nurses to stand for some time, say from thirty to forty hours, when a deposit takes place of white, flaky, pulverulent grumous matter, being the caseum or peculiar principle of the milk formed in the breasts during gestation.'" 56.

The conclusions which Mr. Kane drew from his experiments were the following:—

"That a white flocculent precipitate, similar to that described, subsided spontaneously after twenty-four hours, not only from the urine of pregnant wo-

---

* See Lowder's MS. Lectures. † Elem. Artis Obstet. pp. 46-7.

men, but also in equally great quantity from that of a virgin, ætat. 14, and that of a woman nursing for two months.

That in all the cases of pregnancy the urine was found to contain a small quantity of *albumen** in its uncoagulated state, although this was not observed in the urine of unimpregnated females contemporaneously examined." 57.

### TANGIBLE SIGNS OF PREGNANCY.

They are obtained by an examination, either per vaginam, or of the abdomen and its contents outwardly. The first of these methods, or, as the French designate it, "le toucher," informs us of the condition of the mouth and neck of the womb, which, in pregnancy, undergoes very marked changes, and of the womb itself, in respect to its bulk, its weight, and also its contents, at least occasionally.

We have not space to allude more in detail to each of these particulars, and shall, therefore, merely mention those which are least understood, and which have not been sufficiently explained in some of the more recent manuals of midwifery. The late Dr. Gooch, always a respectable authority, used to insist much upon the importance of employing the vaginal and abdominal explorations at the same time; while the fingers of the right hand are applied upon the neck of the uterus, pressure is to be made upon the uterine tumour, above the pubes, in order to ascertain whether the moving of the tumour above will alter the situation of that felt in the vagina, and *vice versâ*. We cannot, however, properly expect to discover the nature or contents of the tumour in this way; all that is announced to us is, simply, that there is an enlargement of some of the pelvic viscera, or that there is a morbid growth in the pelvis; thus, ovarian dropsy, extra-uterine conceptions, the presence of moles, hydatids, and so forth, in the womb itself, will give rise to this sign, and Dr. Gooch was incorrect in asserting, "that we are, by this means, *certain* that the tumour which we feel is an enlarged uterus."

There is a method of performing "le toucher" which is much recommended by the French writers, and which, when applicable, for it is not in all cases, may assist us a good deal in determining the existence of at least "a something" in the cavity of the uterus. If the finger be pushed against the anterior part of the organ, between the os tincæ and the pubis, with a sort of jerk upwards, we may sometimes so tilt the depending head of the child, as to make it rise up from the finger through the liquor amnii, when it again falls down, with a slight shock or impulse, on the finger. This passive motion of the fœtus, or, as the French term it, "abattement," is, indeed, a valuable sign, when it is distinctly felt; but all the periods of pregnancy do not admit of its application; if the child be too small, as before the fifth month, or too bulky, compared with the quantity of the fluid in which it floats, as after the seventh month, the sensation will, perhaps, not be felt at all; besides, it is possible that a pediculated polypus in the uterus, or a complication of ascites with the presence of some tumour in the pelvis, may give rise to it. As a matter of course, it does not indicate whether the child be

* "It is scarcely necessary to state, that the solution of the bichloride of mercury affords by much the most delicate test for this, a few drops of it throwing down a white flaky precipitate."

dead or alive. So much for the vaginal examination; the abdominal is frequently still more important. Let the woman be laid on her back, with the head and shoulders elevated, and the limbs well drawn up, and then, after a deep and sudden expiration, as after coughing or sighing, the physician is often enabled to feel, not only the contour of the uterine tumour, but even the parts of the child, when the pregnancy is considerably advanced. Having done this, our next step is to discover, if possible, the motions of the child, by the hand applied to the abdomen, and pressing it suddenly, and with a degree of succussion; this causes the child to start, as it were, and give a jerk or slight kick, which is readily recognized in many cases.

If, however, we fail in this way, the ready expedient of plunging the hand first in very cold water, and then suddenly laying it over the abdomen, will often at once detect the movements of the child; these movements, it will be understood, are muscular, and indicate the life of the being which impresses them; but there is another kind of movement, which is the effect merely of mechanical weight, and is produced by the body of the child rolling over from one side of the womb to the other: the mother is often sensible of this, upon any sudden change of position, especially if the child be dead; and when felt by the physician, upon pressing or tapping the abdominal tumour first at one side, and then at the other, it is quite like that of a solid body, falling against the side of a membranous bag, containing a quantity of fluid, in which it is partially suspended. The French have given to this passive motion the appellation of "ballottement." Any moveable tumour, as that from enlarged ovary, complicated with ascites, may give rise to an impulse against the hand of the explorer, which may be mistaken for the true "ballottement."

Before proceeding to detail the audible signs of pregnancy, it will be useful to mention a method of examination which was long ago recommended by Wrisberg, and which, although decidedly useful, has not met with the attention which it deserves. Our author has been much in the habit of employing it, and his experience warrants him to inculcate its utility on his professional brethren. By applying the cheek to the abdomen of a pregnant female, the motions of the child may sometimes be very distinctly perceived, which were extremely faint, or altogether imperceptible, to the hand.

"It is not easy (says Dr. Kennedy) to explain why we should arrive at more accurate conclusions in this way, than merely by the use of the hand; possibly, the weight of the head being irksome to the fœtus, induces it to struggle as it were to free itself from the pressure, by which means it imparts to us, as well through the sense of touch in so delicate a part as the cheek, as through the ear, its slightest motions, which might have escaped our observation on the application of the hand. However, the principal advantage we derive from this means of exploring seems to be, that, by allowing the head to rest for a sufficient length of time on the abdomen, the abdominal muscles are not, in the mere resting of the head on them, stimulated to contraction, as they are in a manual examination; on the contrary, they become fatigued, and being (as we may express it) taken off their guard, they relax and yield, allowing the head to sink more and more into the abdomen; until at length, when there is no gravid uterus in the way, we may often succeed in pressing the cheek upon the vertebral column, thus convincing ourselves of the absence of pregnancy, at least in an advanced stage." 64.

### Audible Signs of Pregnancy.

The application of percussion to the elucidation of abdominal diseases, although as old as the days of Hippocrates, has been but very rarely resorted to by the accoucheur, to assist him in his diagnosis of pregnancy. Its utility indeed, is and must be, from the nature of the varying surface on which it is made, always limited, and often completely nullified; but this is no reason that we should refuse to admit it among our means of diagnosis; knowing its uncertainty, we should rather strive to increase our knowledge of its powers. If we tap upon the hypogastrium of an unimpregnated, or recently-impregnated female, a tympanitic sound (provided the urinary bladder be empty), like that which arises from tapping the blown-out cheek, is elicited; the reason of this is abundantly obvious—the intestines are in contact with the abdominal parietes, and the uterus is still imbedded within the pelvis. In proportion, however, as this organ rises up, and overtops the level of the pubis, the sound which is heard on percussion becomes dull and fleshy, similar to what is caused by striking the thigh with the fingers; the extent upwards over which this dull sound is heard, increasing as pregnancy advances, and the range of the tympanitic sound becomes more and more circumscribed, being confined chiefly to the lateral parts of the abdomen. In the latter months of gestation, the sound, immediately above the pubis, has a drier and harder character than it has hitherto exhibited; this arises from the percussion being applied upon the head of the fœtus, which is usually situated downwards. The seat of this dry (sec) sound necessarily varies with the position of the head, and, as we can not unfrequently distinguish the head of the child by a manual examination through the abdominal integuments, we are thus enabled to corroborate the evidence we arrive at by percussion. Much dependence, as we have already stated, cannot be placed on these phenomena;—far otherwise it is with the other branch of auscultation, when rightly applied to the discovery of pregnancy, at least when it has advanced beyond the first two, or three months.

The author, to whom the merit is due of having first applied Laennec's immortal discovery, to the advancement of obstetrical science, is M. Major of Geneva, who in the ninth volume of the Biblioţheque Universelle, announced, that the pulsations of the fœtal heart might be heard, through the abdominal parietes of the mother, in the latter stages of pregnancy:—But with this simple fact did he rest satisfied, and prosecuted his researches no further. In 1822, Dr. Kergaradec published his "Memoire sur l'Auscultation appliquée à l'etude de la Grossesse" in which, still ignorant of his predecessor's labours, he distinctly proved that not only might the action of the fœtal heart be ascertained by means of the stethoscope at a period much earlier than M. Major had supposed, but also that a whizzing murmur, or souffle, (such as is observed in certain diseases of the heart, and large blood-vessels) accompanying a simple pulsation, synchronous with the maternal pulse, was clearly perceptible at the same time. The cause of the latter sound, he believed to be the circulation of the blood through the placenta; and naturally concluded, that where the sound was heard, the placenta was attached to the womb. Now as this "souffle placentaire," or as Dr. Kergaradec calls it "battement simple avec souffle" may be heard for a considerable period anterior to the earliest recognition of the fœtal pulsation, we shall examine it first. Upon applying the ear over the uterine tumor, either directly, or with the stethoscope interposed, we perceive a blowing, or hissing sound, well known to auscultators, by the name of the 'bruit de soufflet;' sometimes it has more of a rasping, or sawing character; at other times it resembles the cooing of a dove, or the drone of a bagpipe; but these varieties are only occasional; by far the most common being the whizzing murmur. The extent over which it is perceptible is very different in different cases; it may be limited to one spot, which the end of the instrument covers; or it may be heard over the greater part of the uterine swelling; still, as a general rule, we should say that it is at the lower and lateral parts of the womb, where it is strongest and most readily discovered; and the placenta will be found attached to the spot, where the sound had been heard; at least this is true, in a vast majority of cases. Exceptions do occur, now and then, but they are rare, and the general position will be found in practice to be correct.

Dr. Kennedy informs us that at first he was of opinion that the seat of the souffle uniformly denoted, the adherence of the placenta; but that he is now satisfied that a similar sound may be occasionally heard over the lateral part of the uterus, although the placenta is not actually attached to this part. The explanation is given in the following words.

"In the neighbourhood of the ligaments, at the lateral parts of the uterus, we shall also find a more full distribution of vessels, even when the placenta is not attached there, as the principal vessels which connect the uterus with the maternal system pass into it at those points." 69.

Whenever indeed the vessels of a part are in a state of very active and plethoric circulation, we shall discover a certain degree of the bruit de soufflet, upon an attentive auscultation;—it is the case in aneurism from anastomosis; and the very striking resemblance between certain parts of the impregnated uterus, and this morbid structure is well worthy of notice. If we make a section of the uterine walls, at the part where the placenta adheres, or from which it has been recently detached, we discover a congeries of large tortuous vessels, ramifying through its substance, and expanding into cells, or sinuses, which open upon the internal surface;—the rest of the organ exhibiting the usual fleshy-parenchymatous structure, with merely occasional vessels interspersed.

Authors have not however been agreed as to the true seat of the sound, under consideration; and not a few, even of the most recent and eminent explorers, suppose that it resides in the iliac arteries, where the enlarged uterus exercises a compression on their tubes; such is the opinion of M. Bouillaud, as explained by him in a late number of the Journal Hebdomadaire, and which our readers will find alluded to in our last number, page 519. Dr. Haus of Wurzburg advocates the same doctrine, and objects to the placentary origin of the sound, because it is occasionally intermittent, and is often perceptible over the whole uterus.

"In answer to these arguments, it is merely necessary to state, that as to the intermission, the same objection would hold against the supposition that the *souffle* is produced in the aorta or iliac arteries. Again, how does he explain the fact of the sounds being heard in the part of the uterine tumour corresponding to where the placenta is attached, and ceasing when this system of vessels becomes impervious, or the uterus contracted? As to the fact of its being heard over the whole uterus, if this gentleman had sufficiently inquired into the matter, he would have found that it is rarely if ever perceptible over the whole of that organ, although it is often met with over a greater or less extent of its anterior wall; and in such cases he might have detected the placenta attached there: also, where the sound could not be detected, he might have found it attached posteriorly. Further, had he in such cases attended to the position of his patient, and examined minutely and carefully in the lumbar and iliac regions, he would have been less frequently disappointed in his attempts to detect it than he appears by his own account to have been." 73.

The theory of M. Bouillaud, whom we admit to be one of the most accomplished auscultators of the present day, is met by some powerful objections. If the sound be produced by the narrowing of the iliac arteries, in consequence of the pressure of the gravid uterus on the calibre of these vessels, why is the phenomenon not uniformly observed in the case of other pelvic tumors? and why should it cease, as we know that it does, when the fœtus dies, although we had heard it most unequivocally, at an anterior period of pregnancy? Besides how will M. Bouillaud explain the fact, that the sound can be heard sometimes only over a very limited extent, and almost always either at that part where the placenta is attached, or at the side of the uterus, where the chief congeries of the hypogastric and spermatic vessels exists? " These facts," says Dr. Kennedy, " have been repeatedly proved by manual examination, when it has become necessary to introduce the hand into the uterus to remove the placenta, as well as by ocular demonstration after death."

It is scarcely necessary to allude to the hypotheses of those, who have strangely supposed that the placentary bruit resides in some part of the fœtal circulation, as the umbilical arteries; or in any of the venous trunks, whether of the mother, or of the child;—we need only remember that the accompanying pulsations are uniformly synchronous with the beats of the mother's pulse,—a sufficient answer to the first of these theories, and that the stream of the blood in veins, is continuous, and not in jerks, or periodic impulses, as a refutation of the second.

We have next to inquire, what is the earliest period of pregnancy, in which it is possible to detect the placentary murmur.

According to Dr. Kennedy's experience, it cannot be heard before the end of the second, or beginning of the third month;—he has repeatedly succeeded in detecting it in the tenth, eleventh and twelfth weeks.

It is important to attend to this, as we shall find hereafter, that the fœtal pulsations cannot be ascertained, until the 17th or 20th week of pregnancy. The following cases are abundantly illustrative.

" August 15th, 1829. A woman named Devereux, who had been under my care in labour eighteen months before, called to consult me for a slight attack of pneumonia. She mentioned that her menses had not appeared for the last two months: I therefore examined her with the stethoscope, and detected clearly the placental *souffle*, although no uterine tumour was observable. Dr. Collins, who also examined her, expressed his astonishment at its distinctness at that early period. I gave this woman reason to suppose it possible that she was pregnant, of which she had not the slightest anticipation. However, the accuracy of the diagnosis was attested by her coming into hospital on the 7th March, 1830, in labour, and being delivered of a living child the day following, exactly twenty-nine weeks from the period at which we had examined her." 82.

*Case* 2. " Dr. Mollan desired my attendance to examine a patient of his labouring under insanity. She was married, and had been living with her husband about three months before our seeing her, since when, no menstrual discharge had been observed. No further evidence of pregnancy could be arrived at, and the patient was so unmanageable, as to prevent our ascertaining any thing by a vaginal examination; with much difficulty she was kept quiet long enough, to allow the stethoscope to be applied. When the *souffle* was distinguished in the uterine region, but no tumour could be detected, we explained, that it was impossible to give a decided opinion upon this single symptom; but that the impression on our minds was, that she was pregnant at a very early stage; Dr. M. regulated his treatment accordingly, and I three months afterwards learned from him, that there was no doubt of the pregnancy, as he and Dr. Hanna then distinctly detected the fœtal heart and the motions of the child." 83.

*Case* 3. "I was sent for one morning by a lady, who came over clandestinely from the sister kingdom. The statement she gave me was as follows. She had for some months been in the habit of receiving the attention of a gentleman to whom she had formed an attachment; but unfortunately fell a victim to her father's caprice, who, after countenancing this attachment, suddenly withdrew his consent to their union, and insisted on her marrying an individual of his own selection. Ten weeks had elapsed from the time of her first giving way to illicit intercourse, when she consulted me; during which two monthly periods had passed without the usual menstrual discharge: and ten days before my seeing her, in consequence of some active exertion, a discharge of blood took place from the vagina, which lasted for a few days. Her father urged her compliance with his wishes; and she, dreading to enter the marriage state whilst there was a possibility of her being pregnant, consulted a medical man of eminence, who, after the usual investigation, pronounced that such was not the case. Impressed, however, with a painful foreboding of the true nature of her state, although she had no further symptoms of pregnancy than that already mentioned, she determined on obtaining further advice; and, under a pretence of visiting a friend in the country, came over to Dublin. On the most accurate examination, I could ascertain no further grounds for suspicion than the presence of a remarkably distinct *souffle*, which was discoverable on pressing the end of the instrument in the pubic region over the uterus. Relying on this, I gave her to suppose that there was a strong likelihood of her being pregnant, although I could not actually pronounce such to be the case. The result fully justified the confidence reposed in this, as a means of diagnosis, for, exactly nine months from the period when she calculated, she gave birth to a child." 81.

Few readers will be bold enough to impeach the authenticity of these cases, or the strict veracity with which they are reported; and yet, strange to say, not only most practitioners, but even many authors, of the present day presume to treat auscultation with neglect. If they will not use their ears, and satisfy themselves of its truth, they must blame their own wilful and obstinate ignorance if they are frequently perplexed in numerous cases, where they, as well as their patients, are most anxious to obtain a correct diagnosis: we tell them that, if they will but take the trouble patiently, attentively, and repeatedly to listen, either with the naked ear or with the stethoscope, over the hypogastrium of a woman who is with child, they will hear the sound which we have endeavoured to explain in the preceding pages, and the ascertaining of which was of such great importance in the cases we have detailed. True it is that auscultation, like every other new object of knowledge, requires a certain portion of time and of trouble to be able rightly to appreciate its value; and equally true is it, that the student may encounter perplexing difficulties and discouragements in his first essays to acquire the quickness of ear which is necessary to detect its indications. He may mistake other sounds for the one he is in search of, or he may hear the true placentary murmur one day, and not hear it on another; or, lastly, he may completely fail in detecting it at any time, although all the other symptoms of pregnancy be present. A few explanatory remarks may, therefore, be useful. With regard to the sounds which simulate the placentary souffle, they are derived either from the chest, or from the abdominal viscera, or from the aorta, and other large vessels. The common respiratory murmur is sometimes so propagated along the abdominal parietes, that it may be heard by the ear applied over the pubis; equally so is it with the sonorous and other râles; but, independently of the very dissimilar character of these sounds, they are simultaneous with the breathing, and have no synchronous action with the pulse at the wrist, a coincidence which necessarily accompanies the true placental souffle; did we require any other disproof, we have only to examine the abdomen higher up, and the sound will be found to be more and more distinct as we approach the chest. By attending to these particulars, we may also easily distinguish any intestinal rumbling, produced by the passage of air from one portion into another; the noise heard varies in its seat, its intensity, and character, and there is no harmony between its repetitions and those of the pulse. The third set of simulating sounds is much more difficult of accurate discrimination, namely, those which have their seat in some of the large arterial trunks of the abdomen or pelvis; they closely resemble, nay, sometimes are identically the same as, the true placentary murmur, and depending, as they do, upon the same current of blood, they are necessarily synchronous with each other, and with the pulsations of the heart.

"Fortunately (says Dr. Kennedy), such cases are rare, and although we have frequently intentionally produced *bruit de soufflet*, by pressing the end of the instrument on the aorta or iliac arteries, yet, amongst the number of patients examined whilst attending to this subject, we have met with but one case likely to be confounded with pregnancy; where a sound resembling the placental *souffle*, from a morbid cause was observable. The case alluded to was one of considerably enlarged liver, the pressure from which appeared to have this effect; but here the sound was confined to a small spot immediately over the aorta. We shall be enabled to distinguish *bruit de soufflet*, when it so occurs, or arises either from aneurism, hemorrhage, hysteria, or nervous states of the system, by its concomitant symptoms; and in the latter cases, to use the words of Laennec, 'when the bellows sound exists in the aorta, particularly the ventral portion of it, there is always a marked state of disorder in the nervous system, viz. agitation and anxiety, faintings more or less complete, and produced by the slightest causes, and an habitually quick pulse.' "* 77.

The limited extent over which the bruit de soufflet can be heard, and the variableness and irregularity of its strength at different periods, appear to be the chief, although, we admit, not always quite satisfactory diagnostic marks. If, indeed, the compressing cause be a moveable tumour, or an accumulation of hardened fæces in the colon, or the stethoscope itself (for it must always be remembered that a certain degree of this bruit may at any time be produced, by leaning the instrument too forcibly over the canal of a considerable arterial trunk), then we may find it cease altogether when such pressure

---

* Laennec, by Forbes, 2nd Ed., p. 698.

is removed, as by raising the tumour with the hand, or by altering the position of the patient, by clearing the intestinal passages, or by a lighter and more adroit application of the instrument.

But, although we have completely satisfied ourselves that it was the placentary, and no other sound, which was perceptible upon our first examination, it may possibly occur that we may be foiled to hear it at another time. It seems, therefore, that it is sometimes intermittent; and this phenomenon has been so puzzling to explain, that it has led Dr. Haus and other observers to suppose, that it cannot possibly reside in any of the uterine vessels, which, to our knowledge, are not subjected to any periodic changes. It must be frankly acknowledged that, hitherto, no satisfactory solution of this difficulty has been given. Dr. Kergaradec supposed that the intermissions were attributable to changes of position assumed by the fœtus. This may possibly be the case, but the idea, as yet, can be received only as conjectural.

"In such cases, the cessation of the sound is not permanent; therefore, by repeating our examination, we shall succeed at another time in discovering it. Uterine contraction suspends it, in most cases completely, whilst this organ is in action; in some, it converts it, during the pain, into an abrupt sound or pulsation; the former are the cases in which we have most frequently observed this omission to occur." 79.

But now and then examples occur in practice, where not even frequently-repeated auscultation of the abdomen can detect the placentary souffle; perhaps, as yet, we are ignorant of several of the conditions in which it is awanting; but there is one, hitherto little noticed by authors, well worthy of our attention—we mean, when the placenta is attached to the posterior wall of the uterus, and the sound of its circulation is so muffled and obscured by the intervening contents, that it scarce can be recognized by the ear, applied over any part of the hypogastrium. Whenever this state of things is suspected, let the stethoscope be placed over the sacral and lumbar regions, close to the ilium, and we shall sometimes succeed in hearing the expected sound.

Having thus sufficiently explained the earliest auscultatory sign of pregnancy, we proceed now to the examination of the second, which is yet more easy of detection, and more decisive and important as a means of information. The double pulsatory sound of the fœtal heart, if once heard, cannot possibly be confounded with any other, except in those cases where the maternal circulation is very much quickened; and even then, there is a want of synchronous harmony between them. The fœtal pulse generally beats about 130 or 140 beats in the minute; but, like that of an independently-existing animal, it is liable to very considerable vicissitudes in point of frequency.

It is decidedly affected by the motions of the fœtus itself, being usually accelerated after such—by any action of the uterus, especially on the approach of and during labour—by hæmorrhage, venæsection, or any sudden mental emotion affecting the mother.

*Case* 1. A woman was seized with labour-pains, while suffering from a severe attack of croup. The febrile excitement ran high, and the pulse was 140. The stethoscope, applied midway between the umbilicus and the right anterior spine of the ilium, detected the beatings of the fœtal heart, which were weak, indistinct, and much quickened, being 190 or 200 in the minute.

A feeble, delicate child of the 8th month was born, and its pulse then amounted to 180.

*Case* 2. Another patient was seized with violent pleuritis during labour. Her pulse was 140. The fœtal pulse could be heard over a considerable portion of the abdomen, extending across the whole hypogastric region, into the inferior part of the umbilical and lumbar regions ; the number of beats was 180 in the minute. The placental souffle was audible only over a small spot in the left groin; it corresponded in frequency with the maternal pulse.

The patient was largely bled; her pulse rose to 150, and then to 170, and the fœtal pulse fell first to 150, and then to 90. Keeping the ear applied for some minutes to the stethoscope, while the patient was very low, although there was not complete syncope, Dr. Kennedy found that the action of the fœtal heart varied considerably in frequency, being one minute 92, next rising to 100, and then to 128, and that it ranged between the two extremes, until the mother had recovered from the effects of the bleeding; then her pulse fell to 130, and that of the fœtus rose to 135. It now continued between this and 100, the frequency varying every two or three minutes for half an hour, and at last it ceased altogether. The woman was delivered in the evening of a child, which had all the appearances of having recently died.

*Case* 3. A woman was admitted into the Dublin Lying-in Hospital, with uterine hæmorrhage. The placenta was found to be separated from the posterior part of the cervix uteri. She had felt the motion of the child a few minutes before the examination. The placental sound was heard at the left side, stretching into the iliac region, about 100 in the minute; the fœtal pulsations in the neighbourhood of the umbilicus—they were feeble, and 108 in number. The hæmorrhage continuing, the mother's pulse rose to 110; that of the fœtus fell to 88.

"Just at this moment the child was felt moving violently, or rather convulsively, both by the patient and myself. These motions were repeated four or five times in the course of a few minutes, and then ceased altogether, after which the fœtal pulsation could not, upon the closest examination, be detected. The placental sound still continued audible, but became altered and abrupt in the character. The evident inference in this case was, that the child had died from the effects of the hemorrhage, and that the change in the pulse, and convulsive motions observed, were the forerunners of its dissolution. I mentioned this to the pupils who were present with me whilst making the examination, at the same time expressing my conviction that the child would be born dead. In about three hours afterwards, the patient was delivered of a large female child, dead, but exhibiting every appearance of recent vitality, and of having lately been in the perfect discharge of its functions. It was examined six hours afterwards, when the heart and great vessels were found loaded with dark blood, and the sinuses and vessels of the brain similarly circumstanced." 96.

These cases clearly shew the decided influence, which disorders of the mother's system exercise upon the fœtal circulation in respect of its frequency and strength.

The extent over which it may be heard is equally subject to differences; in the majority of cases, it is to be met with over a surface of three, or four inches square, in the hypogastric region, and generally it is more clear and distinct on one side, than on the other; sometimes indeed in the course of the linea alba; and sometimes on both sides, as well as there. In advanced pregnancy, its most usual site, is midway between the umbilicus, and one of the anterior spines of the ileum; even in the earlier periods, this is sometimes the spot, where it can be most easily ascertained. We must therefore carefully explore the whole surface of the uterine tumor, before we pronounce the sound to be inaudible. No doubt these differences in the situation of the sound, depend upon the varying positions of the fœtus. Whenever there is a large quantity of liquor amnii, the sound of the heart is less obvious; perhaps this is one of the reasons, that it is so much less distinct in early, than in more advanced pregnancy:—no doubt, also the feebleness of the little organ contributes to the same effect. It is rare that the fœtal pulse can be detected before the expiration of the fourth month, and until after quickening has taken place; the uterus then rises higher, out of the pelvis, the fœtus becomes more active, and is situated closer to the abdominal parietes than hitherto. At this period, it is always weak and indistinct, and although heard to-day, may not be audible to-morrow; even the lapse of a few minutes will cause the disappearance of all sound, and then it will return; the greatest nicety of ear, as well as patience in renewing the examination, are requisite, in all stethoscopic inquiries, to discover the early fœtal pulse. Dr. Kennedy has met with cases where he has been quite puzzled to hear it, at a first examination, in the fifth month, although the placentary murmur was very distinct; and yet a few days subsequently he detected it at once, in the very same spot which he had before examined without success. In proportion as pregnancy advances, the sound of the fœtal heart becomes more and more distinct, and less subject to intermittent irregularities; the liquor amnii is now considerably reduced in quantity; the child no longer floats about in the water, but remains more steadily in contact with the walls of the uterus. Not only does the sound become more forcible, but the extent over which it may be heard is much increased, so that, towards the full period of gestation, it may be perceived over the greater part of the uterine swelling, and more especially in the lower part of it, between the pubis, and an inch below the umbilicus.

Such, therefore, are the two auscultatory signs of pregnancy—the placentary bruit and the fœtal pulsation; when the former only can be discovered, there may be room for some hesitation in pronouncing upon the nature of the case—where both, or even the latter by itself, is clearly and satisfactorily made out, no rational mind can hesitate a moment. We know that some very recent authors have hazarded an opinion unfavourable to the use of auscultation in such cases; but we are inclined to impugn rather the accuracy of their ears, than the sincerity of their hearts or the sagacity of their heads.

To adduce arguments from mere reasoning, on a subject cognizable by the senses alone, is utterly ridiculous, unless we are to become disciples of the Berkeley school, and boldly deny the existence of all matter, and, therefore, despise all methods of appreciating its qualities. The limited opportunities of some practitioners may, indeed be unfavourable to their ever acquiring that adroitness of examination, and that delicacy of hearing, which are necessary to make a good obstetrical stethoscopist; but surely a public teacher, and a physician of any midwifery institution, ought henceforth to hesitate, before they decide upon the merits of a question which appeals to one of our outward senses as its only fit tribunal. Far better will it be, alike for their candour and their professional character, at once to avow either the cloudy dulness of their perceptions, or the wilful obstinacy of their prejudices.

We consider it to be a duty to ourselves, who were among the first to introduce the knowledge of auscultation to the British public, and have continued to be its firm advocates up to the present hour, as well as to all our readers, especially such as are abroad, and whose opportunities of consulting the mass of modern works are necessarily limited, to seize every occasion of inculcating the importance of employing auscultation, in all cases where it is applicable. Hitherto, its domains have been chiefly the chest, and the courses of the larger arteries ; but, like every other discovery based in truth and nature, the extent of its value can never be duly appreciated at first; its progress increases with our inquiries, and one of its very noblest claims to our favour, and one of its proudest triumphs, is the light which it has so unexpectedly shed upon obstetrical science.

Will any one presume to gainsay its importance in the following cases ?

" I was requested by Dr. Mollan to examine with him a lunatic patient under his care. She was a married woman. For some months before our seeing her, her menses had not appeared. This circumstance excited a suspicion that she might be pregnant; a fact which it was of importance to ascertain, as well with a view to treatment, as to the obvious precautions necessary to have recourse to under such circumstances. She was so perfectly deranged in intellect, that the usual means of arriving at information, through the individual's own statements, were quite unavailable ; and so uncontrollable was she, that a vaginal examination was out of the question. The stethoscope was applied, and in an instant the question was decided, as a fœtal heart's action was distinctly perceptible, both to Dr. Mollan and myself. The motions of the fœtus were also distinguishable in the same way. This woman was some months afterwards delivered of a full-grown child. Had the medical attendant in this or similar cases, from ignorance of the existence of pregnancy, had recourse to treatment calculated to restore the menstrual secretion, what might not have been the result? and yet it is the course that would in all likelihood have been adopted by an incautious practitioner."

*Case* 2. " Mrs. W. mother of two living children, in the third month of her late pregnancy, had considerable hemorrhagic discharge per vaginam, attended with severe pain in the back. These symptoms ceased in a few days, by the observance of strict quiet, joined with the use of the ordinary remedies. She was similarly affected, at intervals of about four weeks, for three successive periods. Between the second and third of these periods, she said she began to feel the motion of the child. After the fourth return, though she declared her unabated confidence in the accuracy of her sensation, as to the movement of the child, I thought it advisable to institute a more strict investigation. On an examination per vaginam, the cervix uteri was not so altered as to remove my doubts, and the 'ballottement' of the French writers never afforded me such undoubted evidence of pregnancy, as a reader of their works might be led to expect. I carefully applied the stethoscope, and distinctly heard the pulsations of the fœtal heart, which fully satisfied me as to my patient's state. She carried the child until about the seventh month, when labour came on, and an infant but very recently dead was born. The recovery was tolerably favourable." 105.

Dr. Byrne has communicated the details of a very interesting case, which had been pronounced by the medical attendant to be one of a cancerous tumour growing from the fundus of the uterus, and involving the Fallopian tubes and ovaries. The treatment usual in cancerous diseases had been employed with no effect. The patient was a pale delicate woman, 34 years of age, and mother of three children, but had not been pregnant for three years, during which time the catamenia had been extremely irregular. She had been much distressed by the growth of a tumour, which seemed to rise from the pelvis, as she was quite uncertain whether she was in the family-way or not. The general symptoms were occasional sickness, pains about the loins, loss of appetite, and absence of menstruation for five or six months. Upon consulting her accoucheur, she was informed of the dismal malady with which she was afflicted. Dr. Byrne, upon applying the stethoscope immediately below the umbilicus, heard a masked murmur, very different from the borborygmus of the intestines; and changing the instrument a little to the right side, he distinctly recognised the placental souffle; on the left side he could hear the fœtal pulsations, which were 140, while those of the mother were only 90. Two months after this examination this patient was delivered of a healthy child.

But if the preceding case offered any rational grounds of embarrassment, how much more annoying and distressing were they in the following one, where even an experienced and skilful accoucheur was quite doubtful whether the woman was pregnant or not? The abdomen was enormously distended with water, and there was general œdema; the dropsical symptoms had existed for a year and a half, during which time the catamenia were very irregular, being sometimes absent for several months, and then returning. When Dr. Kennedy visited her, they had not been observed for six months previous; if pregnant, she had not quickened as at former times; only of late, she thought that she occasionally felt an indistinct motion. No satisfactory information could be obtained by examining either the abdomen outwardly, or the uterus per vaginam, in consequence of the extremely swollen state of every part; but the ear at once discovered the placental souffle on the right side; the fœtal pulsation, although it could not then, was afterwards discovered remarkably small and obscure, but at times it could not be heard at all, whereas the placentary sound was uniformly and easily audible. As the woman was in a state of extreme debility, and suffering from dreadful dyspnœa, it was deemed advisable to induce premature labour. In twelve hours she was delivered of a seven months' child, which lived for 48 hours. The stethoscopic diagnosis in this case was especially valuable, as it had been proposed to perform paracentesis abdominis to relieve the ascitic accumulation. It is quite unnecessary to enlarge the number of illustrative cases; for what medical man, even of a few years' standing, and of limited experience, could not adduce some from the field of his own observation, to prove the extreme difficulty of determining the existence of pregnancy, even within a month or a week of delivery? patients have been physicked without mercy, deluged with draughts and potions, bored through with trocars, and tortured with dismal forebodings, when the small still voice of the stethoscope might have solved every difficulty, and saved the poor sufferer from all her distress; but even supposing that things do not proceed quite so far as this, who does not know the ridiculous and ludicrous exhibitions which sage ladies and grave doctors have in sooth exhibited and do often exhibit? at one time the lady vowing that she ought to be, and that she must be in the condition that dear wives wish to be, when they love their lords, and the poor doctor nonplused himself, not knowing what to think, or how to act, whether to believe her who should know, and kindly to flatter the hopes of the anxious would-be father, or to join in the titter and waggish smile of friends and acquaintances, who regularly send their compliments to enquire how Mrs. —— is, and to know when the doctor expects her to be well. Now there can be little doubt, that the awkward ignorance of a medical man in such a case is but too well calculated to expose the profession to the laughter and comic satire of the world; and we need not be told that unless he acquires and retains the ascendant of a respectful authority, derived from superior knowledge over his patients, never can we hope to see medicine regarded as a justly-noble and useful science, and its votaries treated with honor and submission.

No department of the healing art requires more skill, more caution, more of the gentlemanly character, more of sound information on all subjects than the properly educated accoucheur of the present day; cases of the most perplexing difficulty, involving character, fortune, nay, life itself; and of the most frightful danger and responsibility, are frequently intrusted to his charge; his opinion will, and ought to outweigh the opinion or wish of all others; and his conduct may either save or destroy. Let him not therefore neglect any means of adding to his knowledge, and of supplying him with useful and available information; among such means none promises more important results than the use of the stethoscope. When all the ordinary signs of pregnancy are absent, or so muffled and obscured as to afford scope only for conjecture, if the fœtal pulse can be heard but once unequivocally, the nature of the case is obvious, beyond cavil; the auscultator need not heed all the doubts and discordant opinions of others, for what more can he desire, than to have held converse, as it were, with the very being whose existence is disputed?

There are, however, it must be admitted, sometimes difficulties to be encountered in the path of our research; and just as we have seen above, that the sound of the placental souffle may be confounded with other sounds by the inexperienced observer, so may the sound of the fœtal pulse; the action of the maternal heart, aorta, or iliac arteries, when very rapid, may be mistaken for it; a little attention will however, in most cases, serve to discriminate between them; the simple expedient of invariably comparing the beats of the sound we hear with those of the mother's pulse, will generally suffice; if they do not correspond in frequency, we may be quite satisfied that the sound does not proceed from any part of the maternal circulation, but very strict accuracy here may be rather difficult to obtain, when either the mother's pulse is much quickened, or that of the fœtus is much retarded; in such circumstances we shall be assisted in our diagnosis, by remembering that the pulsation of the aorta, or of the iliac arteries has not the double sound of the heart's action, viz. that which accompanies the auricular, and that which accompanies the ventricular contraction; and moreover, that at each stroke, a thump, or impulse is communicated to the ear, sufficient sometimes to raise it from the instrument, a phenomenon which never accompanies the fœtal pulsation. When the vessel becomes aneurismatic, the throb may be so remarkable as to be felt or even heard by the patient herself. If again the simulating sounds proceed from the heart of the mother, they indeed have the double character of the fœtal pulse; we are therefore deprived of this diagnostic mark; but there is another easily available, and sufficiently decisive; in the one case, the sounds become louder and louder, and the impulse more forcible, as we approach the ear to the cardiac region; in the other, they become more and more obscure, and the impulse is altogether wanting. We select one case to illustrate these particulars.

"Mary ——— came into the Lying-in Hospital in labour. I saw her in company with Dr. Darley; the os uteri was slightly dilated, and the membranes unruptured; she was only in her seventh month of pregnancy. On applying the stethoscope, the true fœtal circulation was no where observed, although there were some circumstances which, with a person not paying attention to the distinctions heretofore insisted upon, might have led to error. The placental sound was heard in this patient to the left side, about three inches from the ramus of the pubis; but it partook more of the character of a pulsation than of the usual *souffle*. On applying the stethoscope to the superior part of the uterine tumour, a distinct double pulsation was observable, beating one hundred and twenty in the minute; however, on feeling the patient's pulse at the wrist, it was found one hundred and twenty also, and synchronous with the pulsation in the uterine tumour. On examining the latter a little more closely, it was found to be the extension of the maternal heart's action, conveyed along the integuments by continuity of surface, and was to be traced distinctly from between the fifth and seventh ribs on the left side, extending on the abdomen, and becoming less distinct in proportion to its distance from the region of the heart, until it was lost altogether just above the umbilicus. The above patient was delivered in a few hours after examining her; the child was dead; and exhibited what are ordinarily termed marks of putrescency." 117.

Such are the chief sources of fallacy, which the action of the maternal circulation may give rise to; and which may thus embarrass the inattentive auscultator;—a diligent caution may easily avoid them. An occasional cause of error is even the sound produced by the contraction of the abdominal muscles, or of the uterus;—it can however be only the totally unpractised ear which is misled in this way.

Supposing therefore that we have satisfied ourselves that the sound heard is that of the fœtal pulse, it may be asked, if it can proceed from no other part, except from the heart itself; Dr. Kennedy answers the question in the affirmative; he states that in some cases the umbilical cord, when placed between the body of the child and the walls of the uterus, may be felt through the thin abdominal parietes, distinctly rolling under the finger, and that if the stethoscope be applied over the place, its pulsations may be detected at once, corresponding in frequency with those of the fœtal heart, and therefore not synchronous, with the mother's pulse;—as a matter of course, this sign is equally decisive of the presence of a living child in utero, as the sounds of the fœtal heart; it has not the double beat of the latter, and it may be rendered less forcible and distinct, by a gradual pressure of the end of the stethoscope over the part; by this manœuvre, a souffle may often be caused, just in the same way, as a souffle may be heard in any large arterial trunk, as the brachial or femoral for example, by resting the stethoscope firmly upon it.

These remarks are well illustrated by the following case.

"Visited Mrs. ——— in an advanced stage of pregnancy; on applying the hand to the abdomen, the integuments were found to be remarkably thin, and the limbs were distinctly to be felt through them. Midway between the navel and pubis, the funis could easily be distinguished, prominent, rolling under the finger, and pulsating; it appeared to be kept in contact with the inner surface of the uterus by being suspended over a limb of the child, and thus pressed between it and the uterus. The pulsation, on the stethoscope's being applied, amounted to one hundred and forty in the minute, corresponding in frequency with the fœtal heart, which was distinctly perceptible in the left iliac region over the ramus of the pubis, and also on the right side, but less distinct. The placental *souffle* was perceptible at the right side, stretching from the neck up towards the fundus of the uterus, emitting eighty sounds in the minute, which corresponded with the maternal pulse at the wrist. What was particularly worthy of attention, however, in this case was the remarkably superficial position of the funis, which rendered its detection by the stethoscope a matter of great facility, and even enabled it to produce a pulsation, which, on careful examination, was perceptible to the touch. Having fixed the funis against the limb of the child, between the finger and thumb of the left hand, I made a gentle pressure with the fore-finger of the right hand on the cord, keeping my ear applied to the stethoscope, the other end of which was fixed over the funis, at a point nearer its insertion into the placenta. The pulsation, which up to the moment of my making this pressure was remarkably strong and distinct, became converted into a *souffle*, and on increasing the pressure it immediately ceased, recommencing the moment I discontinued it. I then removed the stethoscope to the spot where I had discovered the heart's pulsation, and repeated the experiment as above. The action of the heart at first became laboured, but fuller; afterwards it became fluttering and indistinct; and not judging it safe to continue the pressure any longer, lest the child should suffer, I removed it, when the action became regular as before, but somewhat quicker. I had previously placed the stethoscope over the part where the placental *souffle* was observed, but without perceiving any change when pressure was made on the funis as above." 127.

### Compound Pregnancy.

When two children are present in the womb, they are generally so situated that the head of one is towards the cervix, and the head of the other is at the fundus; consequently the exact position of the pulsating points is not the same; and an expert auscultator may hence form a very probable conjecture of the existence of a double pregnancy; in a few cases Dr. Kennedy has been led to this conclusion, and the accuracy of his opinion was confirmed, by the delivery of twins.* It requires however much adroitness in the use of the instrument, and a long practised ear, to be able to pronounce, with any degree of tolerable accuracy, upon this subject; fortunately no very direct advantages are dependant upon, or to be derived from this prophetic knowledge; and even where a physician had reason to suspect the existence of twins, he should carefully abstain from any allusion respecting it to his patient.

In some of the lower animals, as the cat, and bitch, it is often quite easy to determine the number of kittens, and pups they are pregnant with; this

* A most interesting physiological fact has been ascertained in these researches; viz. that the pulsation of the two fœtal hearts, is by no means synchronous; in one case, on the left side, the heart was heard to beat 130 times in the minute, while the other heart on the right side beat 145 times.

arises from the circumstance of the fœtuses being more apart and distinct from each other; our author has never heard the placental souffle, in any, except in the cow. While we admit that in the generality of cases of double pregnancy, it is an object of little moment to know beforehand their true nature, circumstances may occasionally occur, where such knowledge may be of much practical value; suppose that one child has come away, and we are in doubt, whether a second remains in the uterus: instances are on record where the second delivery did not take place for several days, weeks, and even months, after the first; in the case related by Dr. Maton, in the 4th volume of the Medical Transactions, three months elapsed between the births; both children being born alive, and living for some days.

Dr. Ryan informs us of a woman who travelled thirty miles after her delivery; she complained much of swelling of the abdomen; and upon examination, another child was found in utero. Sometimes, although rarely, an abortion of one of the twins takes place, and the other remains behind for four or five months, till it attains its perfect growth, and is born at the full period; now in all such cases, we may arrive at far more decisive certainty of information by means of auscultation, provided the retained child be alive, and sufficiently developed, than by any other method hitherto followed.

### Complicated Pregnancy.

This term is employed by the French authors to designate the co-existence of disease, either in the uterus itself, or in some of the adjacent structures, with pregnancy; thereby rendering the discovery of the latter state obscure, and often most perplexingly uncertain. The most experienced accoucheurs have been embarrassed; what between the reports and assurances of the patient, the enormous distention of the abdominal parietes, thus precluding any satisfactory examination, and the variable character of the symptoms, which one day simulate those of gravidity, and on another day are all referable to an accumulation of wind or of water, it may be next to an impossibility, accurately to pronounce upon the true nature of the case. Suppose, for example, that there is an inordinate quantity of the liquor amnii, so distending the uterus, that it occupies almost the whole abdomen, or that the uterus is filled with air, or that a dropsical effusion has taken place into the bag of the peritoneum, or that the intestines become blown out with air, or that this air is collected exterior to them, constituting the disease of "tympanitis abdominalis," or lastly that along with pregnancy, there is diseased enlargement of one of the ovaries, a complication indeed rare, but occasional, it may defy the nicest tact of the fingers, and the shrewdest sagacity of multiplied experience, to say positively whether our patient be in the family-way, or not. If tympanitic inflation be the cause of the perplexity, we may indeed observe that the size of the abdomen varies much at different times, that it is more equally diffused, and often more conspicuous in the epigastric and umbilical regions, than over the site of the uterus, that the belly on percussion gives out a sonorous drumlike sound, and if examined with the ear, a loud intestinal rumbling, or borborygmus may be heard. In such case, we shall do well to premise a dose, or two, of a warm purgative, and none, according to our author's experience, is so efficacious, as a mixture of castor oil and spirit of turpentine; after a free evacuation of the bowels, the stethoscope may now discover with facility the placental souffle, or fœtal pulse.

When pregnancy is complicated with ascites, the treatment is much more difficult and precarious; but all will depend upon the accuracy of the diagnosis we have formed. In the name of humanity, let no more murders from ignorance be committed. We have already alluded to one case, where a practitioner was about to perform paracentesis abdominis, while ignorant of the co-existence of pregnancy. Sir A. Cooper has reported a similar case; and Dr. Lowder mentions one, in which the trocar was actually plunged through the distended urinary bladder, and uterine parietes, into the head of the child!* But even when cutting and boring instruments are not employed, much and lasting injury may be committed by the exhibition of drastic purging and diuretic medicines during the state of gestation; whereas if the physician were assured of the existence of this, his practice would be more rational, and far more successful; by gentle remedies he would endeavour to evacuate the water, or at least to prevent it from increasing; and were paracentesis indispensably necessary, the operation might be done, not only with perfect safety, but with success. Our limited space forbids us from expatiating at a greater length upon this part of our subject, and from alluding here to some other complications of disease, which may exist simultaneously with pregnancy, and mask or conceal all the ordinary symptoms, which indicate such a state. This we regret the less, as we shall be led to the consideration of some of these in the following section, which treats of

### PSEUDO-PREGNANCY, THE "GROSSESSE APPARENTE, OU FAUSSE" OF THE FRENCH AUTHORS.

Under these terms are comprehended all cases of actual deception and of wilful simulation, or pretence, as to the existence of pregnancy, when that state does not exist. It will be useful to consider this question in a threefold point of view, according as there may be, either some physical and appreciable phenomena, in the body of the female, calculated to mislead her, as well as her attendants; or on the other hand, mere longing whims, and foolish fancies, which like the delusions of insanity take and keep possession of the mind of the invalid, in spite of all reason,—or lastly an intentional forgery of some of the symptoms, for the purpose of imposing upon credulity, or of evading the retribution of avenging justice.

In the first class we may enumerate ascites, tympanites, ovarial enlargements, moles and other growths within the womb, amenorrhœa, and hysteria, &c. The morbid developments of the ovaries are among the most frequent and perplexing of these simulating diseases; there is such an intimate sympathy of action and of organic, or vegetable sensibility between these organs and the womb, and between this centre of the female generative system, and the rest of the animal frame, especially the mammæ and stomach, that affections of the one often induce, and still more often imitate those of the other.

---

* Dr. Gooch in his excellent work on the Diseases of Women, tells us of a patient who was taken to the operation-room of a well known hospital for the purpose of being tapped, to evacuate a supposed ovarian dropsy; fortunately she was remanded for further examination, and before the operation day she brought forth a child.

Our patient shall have the morning sickness, the dark-encircled eye, the tumid breast, the distinct areola round the nipple, the full stomach, the absence of her monthly indisposition, and often, too, the feelings of something moving within her, and yet, alas, the true cause of all these delusive symptoms may be the early growth of an incurable disease. Medical men cannot be too cautious in these cases. We are told by Valentin, that at Paris, in 1718, the Demoiselle Famien had a charge of pregnancy and child-murder brought against her, and the issue proved that she was merely labouring under ovarian dropsy. It is not our intention to enumerate the ordinary distinctive symptoms of this disease, for they are well explained in all the common text-books of midwifery; but as our leading object, in the present article, is to illustrate the grand importance of auscultation in a variety of obstetrical cases, we shall confine our remarks on the diagnosis of ovarian enlargement, to the signs which are appreciable by the ear. As a matter of course, no fœtal pulsation can ever be heard, for there is no independent circulation within the abdomen; not so, however, with the absence of all souffle, or whizzing pulsatory sound. Those readers who have perused attentively the preceding pages, and the interesting Memoir by M. Bouillaud in our last Number, will be prepared to understand, how any tumours in the belly may give rise to the production of certain blowing, sawing, or rasping sounds from the adjacent large arteries—they will remember that, whenever a moderate pressure is made over the trajet of any considerable artery, a bruit is heard with the stethoscope. Such, therefore, is the explanation which may be given of the souffle, which is sometimes heard at certain parts of the abdomen, when any abnormal growth is pressing at all upon the aorta or iliac arteries. Our object must, therefore, be to remove, if possible, the pressure, while we are listening: this may occasionally be done by examining our patient in different positions, or by tilting up or pushing to the side the tumour, so as to dislodge it, for the time, from its usual place. If the bruit disappears upon employing any of these manœuvres, then we have reason to suspect the extraneous cause of the phenomenon, or at least our suspicions as to the non-uterine seat of the disease receive confirmation. The longer, too, such a state of things as usually accompanies ovarian disease has existed, the more probable will be the inference, that it is altogether independent of pregnancy; and the farther advanced in life our patient is, the greater will be the chance of the deception. But of these advantages we are quite deprived when the case occurs in a young girl, who, perhaps, has exposed herself to the chance of impregnation—in whose abdomen no distinct or isolated swelling has ever been perceptible, and in whom the only well-marked symptom has been the absence of the menstrual flow. An example from our author's experience will illustrate the subject better than any category of our remarks.

"Catherine ———, ætat. 18, an unmarried girl, was sent into the Lying-in Hospital by the directions of an eminent surgeon in the city, by whom she was pronounced pregnant, and in active labour. On paying the evening visit to the labour ward, our attention was attracted by the vociferations of this patient, and the apparent violence of her labour, in which she outvied all the patients in the ward, several of whom were near being delivered at that moment. On pressing the hand over the abdomen, it appeared distended and tense, but the limbs, or body of the child could not be distinguished through the parietes. This circumstance excited some suspicion; and on making a vaginal examination, the os uteri was felt high, and placed so nearly beyond reach, that we could not with certainty pronounce as to its enlargement. The stethoscope was now applied, when no placental or fœtal sound could be any where detected, but the intestinal murmur was evident over every part of the abdomen. On resting my cheek on the parietes, and allowing it to remain there for some time, as the abdominal muscles were in violent spasmodic action, they became gradually fatigued and relaxed, and the spine was distinctly perceptible without any uterine tumour intervening. After purging this patient freely, a copious menstrual discharge set in, which, with a free evacuation of fæces and wind from the bowels, reduced her abdomen to its natural state, and left not a vestige of pregnancy. The account this girl gave of herself was, that her menses had ceased for some months, since when she had been subject to constipated bowels and occasional sickness of stomach; that she had suffered from slight attacks of abdominal pain with spasm, at each return of the period when her menses should have appeared, but that these symptoms had set in with such violence the day before her admission, as to lead those about her to suspect that she was in labour, an opinion which, as we have seen, was corroborated by the medical gentleman who saw her. This spasmodic action of the muscles, attended with abdominal pain, in a great degree resembling aggravated colic, is by no means an unfrequent accompaniment of amenorrhœa, as several such cases have occurred to me. This possibly may have been the cause of loss of character, in more than one instance. Free purging, with the hip-bath, and in very aggravated cases the use of the lancet, is the practice we have had recourse to. In the case of a maid servant, seen some months since, the details are almost identical with those just mentioned, and there was considerable difficulty in convincing the girl's mistress that she was not in labour, when this spasm and pain set in; more particularly, as she had previously a strong suspicion of the girl's being pregnant, from the amenorrhœa and sickness of the stomach under which she suffered." 168.

At the period of the usual cessation of the catamenia, between the fortieth and fiftieth years of age, many females allow themselves to be imposed upon as to their actual condition. Unfortunately for their own sakes, the mere hope and wishing are often the original grounds of their expectation; and the human mind is of that pertinaciously self-creating, self-deluding character, that if once permitted to dwell upon a mere dreamy vision, it soon will turn fancy into reality, and will persist in embodying its own phantom figures. A cautious and experienced physician will always be somewhat reluctant to assent to the fond imaginings of ladies, at a certain period of life; he will generally find that the menses have been irregular in their return for a much longer time than the supposed period of gestation—that they have been absent for several months occasionally—that then they returned either very profusely or very sparingly; similar irregularities may be also observed in the functions of the stomach—the morning sickness has been felt, perhaps, every morning for some weeks—it then leaves, and resumes its annoyance after some time; besides, it is very apt to intrude itself at other times besides the morning, and often it is accompanied or followed by a relaxed state of the bowels, after the free evacuation of which our patient is vastly better, feeling light, comfortable, and cheerful. In short, there is a general languor and inactivity of the abdominal and pelvic viscera—they seem to be oppressed with a slow-moving circulation, and probably there is venous congestion, especially of the liver, spleen, and uterus. However this may be, true it is that, generally, a very few brisk, warm purgatives, to act vigorously on the bowels and urinary organs, with total abstinence from malt liquors, will often effect a most miraculous improvement, although they cruelly disappoint the hopes of the longing parents, who, like the old Commodore Trunnion and his affectionate spouse, see them all dissipated in an astounding tornado, or cataract of gushing water ! !

But we must not carry our incredulity too far, and set down every case of supposed pregnancy, in rather elderly females, to the mere phantasies of the mind. There would be no difficulty in adducing a number of cases of genuine conception and utero-gestation after the forty-fifth, fiftieth, and even a year or two later ; and there sometimes appears almost an effort on the part of the system to be young again, just before the period which is usually called the change of life.

There remains one other form of disturbed menstruation, which often simulates, for the time at least, some of the phenomena of pregnancy. When menorrhagia is attended with severe forcing pains in the abdomen and back —when clots and apparently-organized membranes are discharged, the ready suspicion is, that an abortion has taken place ; and frequently it is a matter of not a little difficulty, to persuade the friends of the female that the symptoms are not those of interrupted pregnancy, and that the expelled substance is not a regular birth ; but let not any medical man be ignorant of the possibly-true nature of the case, and whenever there is room for doubt, his sacred duty ought ever be, to lean to the interpretation of charity. If such and such occurrences have happened to the observation of others, why may they not equally present themselves to ours ? Morgagni was one of the first authors to point out, that the unimpregnated uterus will sometimes form on its inner surface an organized layer, which, assuming a triangular form, correspondent to the figure of the womb, may be subsequently expelled with labour-like pains. Even moles, or those fleshy fibrous formations which used to be considered as invariably the result of blighted ova, are occasionally developed within the virgin uterus ; and their expulsion may be attended with profuse hæmorrhage and with severe suffering. The possibility of such an occurrence ought never to be absent from the mind of a medical man, and the more imperative is the duty, because almost all the symptoms of pregnancy may be present during their formation. The same remark is applicable to the existence of hydatids within the uterus ; most of the cases, indeed, of these singular developments are reputed to be cases of pregnancy, and their true nature is detected only when they are thrown off ; and the mistake is the more readily committed, as by far the greater number of cases occur in married females, and the vesicular masses are often co-existent with a blighted ovum.

Probably most of the cases of hydrometra, or hydrops uterinus, occurring in the unimpregnated uterus, are really and truly of a hydatic nature, one large vesicle occupying the whole of the cavity. An interesting example of this rare disease is thus detailed.

"It occurred in the person of a confidential attendant of Lady ——, whom I was desired to see by the late Dr. Evory. She was reported to be in a very dangerous state, from a labour of nearly three days' continuance, which had not then terminated. I found her exhibiting all the appearance of a woman worn out with long continued and unavailing labour, her pains recurring at irregular intervals, and she herself much exhausted by the force and exertion used when they were present. Having passed my hand over the abdomen, it did not give the idea of that of a woman in tedious labour, as, although it was certainly very much distended, fully as much so as that of a pregnant female at the ninth month, yet the body or limbs of the child could not be discovered. The swelling was circumscribed like that of an enlarged uterus, and an obscure fluctuation was observed. On examining by the vagina, the os uteri was found undilated, but the neck evidently developed; and fluctuation was perceptible here, although, on tilting up the uterus, no *abattement* could be distinguished. This patient's limbs had been slightly œdematous a short time before, but the œdema had disappeared. I directed her to be put into a warm bath, and gave her some calomel and jalap, which operated in a couple of hours. Whilst she was straining at stool, a sudden discharge of a reddish-coloured watery fluid poured from the vagina; and, on introducing my finger shortly after, I found the os uteri very slightly gaping, and the fluid passing freely from the opening. The uterus felt flabby for some time, but afterwards contracted and descended into the pelvis. No solid substance whatever came away, although a discharge somewhat resembling the lochia kept up for some days. Foderé mentions a somewhat similar case (vol. i. p. 476), in which a young woman was accused of infanticide from this cause." 179.

It is not uncommon to find an immense accumulation of water, with a small blighted ovum, in females whose constitutions have been tainted with the syphilitic poison; but, as a matter of course, such a case is widely different from that we have just reported. Having thus shortly glanced at some of the more common causes of the first class of pseudo-pregnancy, viz. of that in which the patient and her friends are misled by actual or bodily deceits, our attention is next drawn to those curious cases, where the deceit or illusion resides only in the brain of the individual herself—when she thinks, and will think, and is resolved to think that she is pregnant, although, perhaps, not one symptom of that state ever has been present. Not unfrequently, indeed, are the catamenia irregular, or even absent, during the time, and then this very defect may give rise to certain feelings in the female breast, which prompt her to believe that she has conceived; and if the patient be hysterical, and be subject at times to flatulence, or to abdominal pulsation, we can, without difficulty, account for some of her strange vagaries; but then such a case belongs rather to the former than to the present section. An exquisite case of the *genuine imaginary* and *purely ideal* pregnancy is given by our author.

"Mary Connor, ætat. 32, who has had two children, the last nearly nine years since, supposes herself pregnant, in which state she has been according to her idea for the last seven years. She has no symptom whatever of pregnancy, not even enlargement of the abdomen; she says she quickened at the fourth month, since which she has constantly felt the motions of the child up to the present period. States that, at the expiration of nine months from the time at which she became pregnant, symptoms of labour set in, which lasted for three days, and went off again; that at the expiration of eighteen months, she was again attacked with labour, and so on every nine months until within the last two years. Her menstrual discharge has continued to occur regularly every third week since the nursing of her last child. She called on me for the purpose of being delivered by instruments, for which she is very anxious, as she says there is no chance of her being delivered without them. My endeavours to convince her that she was not pregnant were of no avail, as she preferred a similar request to another practitioner, who afterwards informed me of it." 182.

Our readers will find an account of a yet more curious case, in a former Number of our Medico-Chirurgical Journal, for Sept. 1824.

Perhaps the most feasible, and certainly the most easily-applicable, mode of explanation of such singular fancies, is to refer them to a sort of partial insanity, or of monomania; they most assuredly seem to be the offspring of a disturbed, and not of a healthy mind; and if by the terms partial insanity, or monomania, is only meant, an aberration of reason on particular subjects, while the faculty is sound upon others, we cannot refuse to class the fond delusions of women, of which we have been speaking, among the catalogue. Whether phrenology be true or not, it will not be denied that the phenomena of such cases may be accounted for, more easily upon its principles, than upon those of any other psychological system: according to its tenets, the organ of philoprogenitiveness is morbidly active.

### Simulated, or Pretended Pregnancy.

The motives of women, in wilfully, and with perfect knowledge of the deceit which they are practising upon others, feigning themselves to be pregnant, are various; sometimes they are prompted to it by mere caprice, by vanity, or by the desire to regain the estranged affections of their husbands; at other times, it is one of the many tricks of begging to extort alms, and modern history has shewn us that religious fanaticism will borrow its aid; and lastly it is not unfrequently attempted by condemned criminals, as a plea in bar of immediate execution.

It may be worth while to allude for a moment to the notorious case of Joanna Southcote, who managed to play her cards so cleverly, that she actually persuaded a number of medical men to vouch for the truth of her ludicrous lie. Dr. Reece was quite as satisfied of Joanna's pregnancy, as Joanna was herself; and what grounds of belief are more rational, than those of our senses? Dr. R. had felt the motions of the child!! Dr. John Sims had not it would seem, such a delicately discriminating tact in his fingers; for not only did he not feel the child to move, but he presumed to announce in the public papers, that Joanna's big belly contained no child at all. It must be remembered that Joanna's chaste coyness was so sensitive against any rude curiosity of men, that she would never allow a vaginal examination; her warning spirit had commanded her not to submit to such an indecency. Well, year after year passed away, and no promised Shiloh appeared; the inspiredly-pregnant lady began to droop and languish, and little wonder was it, for already did she number 64 years of age. So full of faith were her disciples however, that they, like good Mussulmen, doubted nothing, and the learned Doctor having some idea of performing the Cæsarian operation, asked her whether in the event of an attack of apoplexy, he should not instantly extract the child in this way. [It was indeed a pity that my father Mr. Robert Shandy was not alive, for doubtless he would have attempted to convince Joanna, that gastrotomy is the only method, by which a child can possibly be delivered, without any injury to the delicate network of the brain!] As it was, Joanna did not approve of the proposal, when mentioned by Dr. Reece, and the prophetic physician's hopes, of being the arch-actor upon so splendid an occasion, were irretrievably dissipated.

Upon dissection, it was found that the tumor, which had been mistaken for the impregnated uterus, was the urinary bladder, which Joanna had the power of keeping distended much longer than ordinary mortals can do, and that the uterus itself was actually smaller than it is usually even in the virgin state!!

The last and most important set of cases remains yet to be mentioned; we allude to those in which the unhappy criminal pleads the existence of pregnancy, to stay the immediate punishment of her guilt; and awful indeed is the responsibility which is incurred by those, who are appointed to decide upon the truth, or falsity of the averment. The woman swears upon her oath that she is with child; it may be a wilful lie, or it may not; she may have reasons to think that she really is so; and difficult in all, and utterly impossible in some cases, is it to disprove these beyond all doubt. We have seen by the preceding pages, that in other instances of alleged pregnancy, we invariably avail ourselves of the account which our patients give of their state and of their feelings, and rarely will a medical man presume, to pronounce upon its existence unequivocally, when the female, who may have borne children before, has no suspicion of it. Equally reluctant must he ever be, to assert boldly its nonexistence, during the early months, if his patient believes and reports otherwise; in such a predicament is he situated, when called upon for his opinion, as to the condition of the convicted criminal; he has the solemn oath of the party staring broadly before his eyes, and hardy indeed must he be, who without any wavering will assert its utter falsehood. We do not mean by these remarks to deter the physician from intrepidly doing his duty; we do not advise him to speak against his conviction, even for the sake of that gentlest virtue 'mercy;' justice has a higher claim than humanity, and truth must be preferred to compassion; but we earnestly adjure him, to ponder the subject long and well, to give the benefit of every rational doubt to the prisoner, and only upon the clearest, and most irrefragable evidence, to dare to stamp a negative upon the wretch's affirmation. The following case is adduced to shew the exceedingly painful duty which is sometimes imposed upon a medical man, and at the same time as an illustration of the murderous ferocity of our law in this particular. The case occurred to Dr. Franklin of Limerick.

"March 16th, 1831, Margaret Mackessy, ætat. 35, wife of Edward Mackessy of Aharoulk, was tried before the Honourable Baron Pennefather, for the wilful murder of Mary Mackessy, her mother-in-law. It was a case of circumstantial evidence. The principal witness for the prosecution was Honora Mackessy, a little girl of eleven years of age, the niece of the prisoner's husband. The ill-fated woman was, after a most patient and deeply interesting trial, found guilty of the crime, without the least hesitation, by the jury: and the learned Baron sentenced her for execution on the succeeding Saturday, and her body for dissection. After sentence was pronounced by the judge, the unfortunate woman pleaded pregnancy, and his lordship directed, that a practitioner of midwifery should be at once procured, when I was sent for, and directed by the court to examine the convict; his lordship at the same time, laying down as the law, that *pregnancy alone* without *quickening* of the *child*, would not be sufficient ground for staying the execution, and directing me to examine minutely as to both these points. The convict was removed from the dock to a room adjoining the court, where I examined her. She admitted to me, that she had been suckling a child to within a month or six weeks of her being sent to prison. That she had menstruated once after she had weaned the child, and that from the time she had so menstruated, to the day of her conviction, it was near two months.

"This was merely her own assertion; but though I minutely questioned and examined her on the signs and symptoms of pregnancy, she refused to give the required answers, and merely contented herself with the assertion, that for two months she had not menstruated; but at the same time she admitted she had not quickened. Although I gave her the benefit of her assertion, still upon a careful examination of the abdomen, I was clearly satisfied that she had not quickened. She was then reconducted to the dock, and after being sworn, I was examined by Baron Pennefather ; when I stated my opinion to be, that though the convict might be young with child, which was very doubtful, as it was supported solely by her own declaration, that I was of opinion she had not quickened ; and further added, that this opinion as to quickening was confirmed by the admission of the convict herself. Under these circumstances the Baron did not stay her sentence, and she was executed on the 19th. I waited with much anxiety the result of the *post mortem* examination, which was conducted the same day at the County Infirmary, in presence of six professional gentlemen, when it was ascertained beyond all doubt that she was not pregnant." 191.

Here then is an instance of the solemn interpretation of the law of this land, by a judge upon the bench; of the law which, to use the forcible language of our author, condemns to death a child in the fifteenth week of its existence, while a child in the sixteenth (this being the usual period of quickening) is saved from such an unjust and unmerited fate ! ! The thing indeed is monstrous; and tenfold more monstrous now, than at the time of enactment; for then it was the common belief that fœtal life did not commence until quickening ; but no such apology can be alleged for legislators of the nineteenth century; and yet not ten years back, the following enactment was guided, in the distinction of its clauses, by an utter falsehood. The 43rd section of the 9th Geo. IV. c. 31, provides—

" That if any person, with intent to procure the miscarriage of any woman, then being quick with child, unlawfully and maliciously shall administer to her, or cause to be taken by her, any poison or other noxious thing, or shall use any instrument, or other means whatever, with the like intent, every such offender shall be guilty of felony, and being convicted thereof, shall suffer death as a felon : and if any person, with intent to procure the miscarriage of any woman, not being or not being found to be then quick with child, unlawfully and maliciously shall administer to her, or cause to be taken by her, any medicine or other thing, or shall use any instrument or other means whatever, with the like intent, every such offender, and every person counselling, aiding, or abetting such offender, shall be guilty of felony, and being convicted thereof, shall be liable, at the discretion of the court, to be transported beyond the seas, for any term not exceeding fourteen years, nor less than seven years, or to be imprisoned, with or without hard labour, in the common gaol or house of correction, for any term not exceeding three years ; and if a male, to be once, twice, or thrice publicly or privately whipped, if the court shall so think fit, in addition to such imprisonment." 265.

The meaning of the words " quick with child" are settled by a decision of Judge Laurence in the case of Rex *v.* Phillips ; after hearing the evidence of the medical men, he decided that a woman could not be considered quick with child, until *' she had felt'* the child alive and quick within her ; Whose assertion then can be taken, but that of the culprit alone ? Need we say a single word to shew the worse than Vandal ignorance, and criminal recklessness, of those who uphold the present law ? But, as if reason was laughed at in the framing of some statutes, and as if legislation and jurisprudence were surely the antipodes of common sense and consistency, the strange anomaly has been perpetrated, that by the law of real property an infant ' en ventre sa mere' may take an estate from the moment of its conception, and yet be hanged four months afterwards for the crime of its mother. It is however but justice to acknowledge that the same rigorous interpretation of the law is not always acted upon, as in the case, at which

Baron Pennefather presided. A woman was tried at Carlow, on the 19th of March, 1830, before the Chief Justice of the King's Bench, for the murder of her husband, and found guilty : she pleaded pregnancy in stay of immediate execution, and a jury of matrons was impanneled to try the truth of her plea. The result of the deliberations of these matrons! (some of whom were unmarried, and not one of whom had ever attended a case of labour) was, that "they could give no opinion on the subject—some of them considering that the culprit was, others that she was not with child." Fortunately alike for justice and humanity, Drs. Porter and Byrne were directed to examine the woman. She stated that she had quickened ; this they did not believe—but they admitted that there were sufficient grounds to justify the suspicion of pregnancy, advanced probably to $1\frac{1}{2}$ or 2 months. Knowing, however, the extreme uncertainty of any data, they declined to swear on the subject, and suggested to the Judge, that the prisoner should get the benefit of their doubt. In this, he humanely concurred with the physicians, and granted a respite for a time. This woman was delivered of a male child on the 10th Sept. and died on the 22d of October following in jail.

The preceding case, among others, is a memorable instance of the beautifully lenient and enlightened spirit of English law. On a subject involving life and death, surrounded with difficulties and enveloped in uncertainty, the arbitration rests with a jury of twelve women—women, too, taken sometimes from the lowest dregs of society, such as frequent the purlieus of a court of law, and are akin to the witnesses "with the straw in their shoe," ready to say any thing—ready to swear to any thing! Let us hope that this blot of ignorance and of inhumanity may soon be wiped from our criminal code, and that the same virtuous and distinguished zeal which prompted three of our medical brethren, at a late assizes in Norwich, to step forward to proclaim the ignorant falsehood of the jury, and the iniquity of the verdict, may animate the breast of every member of our profession. A woman is condemned, at Norwich, to death on the 3d of March—she pleads pregnancy in arrest of the execution: 12 matrons are directed to try whether she is pregnant with a "*quick*" child. After an hour's deliberation, they decide that she is not "quick with child," and the prisoner is, therefore, ordered for execution. Messrs. Scott, Crosse, and Johnson, eminent surgeons of the place, here nobly interfered; they examined the woman, and found her not only pregnant, but actually quick with child. Baron Bolland, the presiding judge, immediately reprieved her until after the delivery. On the 11th of July, she was delivered of a large healthy female child. Before leaving the interesting subject of the propriety, or not, of ever involving the life of an innocent being in the guilt of its mother, we are tempted to lay before our readers the following extract from the notes appended to the present work, by an eminent barrister, Mr. Smith. The suggestion appears to us to be an exceedingly good one, or, at all events, it would effect a mighty improvement on the present existing enactments of our statutes.

"If, however, any given period of pregnancy must still be fixed upon as determining the guilt of the offender, it would, perhaps, be more consistent with sound sense, to fix that period at the end of the seventh month of pregnancy, and to punish with greater severity the attempt to expel the fœtus from the womb, at a time when it is not capable of individual life, than when greater development and growth have rendered its separate existence possible. Such a distinction, at all events, would afford surer grounds for a satisfactory conclusion, than the uncertain test which has been adopted in the statute." 267.

There is still one other case, in which a woman may intentionally feign pregnancy, and that is, when the succession to property is concerned. On the death of her husband, or of any other testator who may have left a legacy to her offspring, and, failing that, to the next heir, or to a charity, she, conscious all the time of the deceit which she is practising, may assert that she is with child, in order to gain time for the perpetration of some other fraud, as, for example, the substitution of a child not her own. The law, however, steps in here, and supplies a remedy; the next heir, whether he be heir-at-law, or merely a devisee for life, or intail or in fee, may obtain the issue of a writ "de ventre inspiciendo," for the purpose of inquiring into the truth of the plea. The writ is granted by the Court of Chancery, and issued to the sheriff, who summons a jury to ascertain the reputed pregnancy; and if a verdict favourable to the woman be returned, means are taken to prevent all male access until the period of pregnancy has expired, when, if the plea prove to be unfounded (forty weeks, according to Britton, being the longest time allowed; for, after that period, the pregnancy is declared illegitimate), the next heir is entitled to demand immediate possession of the property; and among the old lawgivers, severe penalties were even inflicted on the offending female.*

It is important to know that, in an inquiry under the writ "de ventre inspiciendo," whether or not the female be pregnant, the result does not depend upon the fact of quickening having taken place; the sheriff is directed merely to cause the woman to be examined, and to return "utrum impregnata sit, necne;" and the fact, that the writ commences by assigning as a reason, that "ipsa falso dicit se esse pregnantem cum non sit," seems to shew, that the mere declaration of the widow herself would not be sufficient evidence of pregnancy, if unsupported by any circumstances, whence their probable truth or falsehood might be ascertained.

We must now draw to a close, leaving a few subjects of Dr. Kennedy's book unalluded to. It is one of sterling merit, of great practical utility, and better calculated to enforce the general adoption of auscultation, in obstetrical pursuits, than any work which has issued, either from our own or from the foreign medical press.

It is, indeed, gratifying to remark the zeal, the industry, and the high professional ability which so much distinguish many of our brethren in Dublin. Dr. Kennedy has proved himself one of the very ablest among them, and has conferred a great practical benefit on the public at large, by the publication of his excellent observations.

---

* The direction contained in the writ is in the following words:—

"Tibi præcipimus quod assumptis tecum duodecim discretis et legalibus militibus et duodecim discretis et legalibus mulieribus de comitatu tuo, in propria persona tuâ accedas ad præfatam R. et eam coram præfatis militibus videri et diligentur examinari et tractari facias pubera et ventrem in omnibus modis, quibus melius certiorari poteris utrum impregnata sit necne. Et si ipsæ mulieres ipsam R. impregnatam invenerint: tunc diligenter inquiras ab eis de tempore quo ipsam crederint fore paritura. Et inquisitionem quam inde feceris, scire facias justiciariis, &c."

We had nearly finished our review of Dr. Kennedy's book, when the very able and interesting paper on the Signs of Pregnancy and Delivery, by Dr. Montgomery, Professor of Midwifery to the King's and Queen's College of Physicians in Ireland, came to hand. Our notice of it must, therefore, necessarily be a brief one; and, to avoid all repetition, we shall confine our remarks chiefly to two very important, and still very obscure topics, which well deserve the minute attention of every obstetrical physician—we mean, the history of what are usually called moles, or false conceptions, and that of a structure which has given rise to even a greater contrariety of sentiment—the corpora lutea of the ovaries.

### MOLES, OR FALSE CONCEPTIONS.

The term mole has, unfortunately, been applied to a variety of formations, differing widely and essentially from each other, not only in their appearances and texture, but also in the causes which have produced them. Had but a proper anatomical investigation been employed in the inquiries of medical men upon the true nature and origin of these bodies, the confusion and uncertainty which have hitherto, and do still perplex us, might have unquestionably been, in a great measure at least, removed. A blighted ovum, the remains of a placenta, masses of coagulated and apparently-organized, blood, and even sarcomatous and other tumours of the womb, have all been classed under the appellation of "mole."

It cannot, then, be surprising that physicians have differed much in their opinions, whether these formations are in all cases consecutive to, and the results of, impregnation, or whether they may be developed within the virgin uterus. The weight of trustworthy medical authority leans certainly to the former of these opinions; but, while it is admitted on all hands, that not only coagula of blood, by remaining for a length of time in the womb, may assume a globular form, corresponding to the containing cavity, and may gradually lose all the colouring particles, so that nothing but a fibrous texture remains, but also that the unimpregnated organ throws off, under certain circumstances, membranous formations, it will require the utmost caution, as well as an intimate knowledge of the structure and appearances of other uterine contents, to pronounce decidedly, in all cases, whether they be truly the result of conception or not. If, indeed, any real remains of the embryo itself, or of its placenta, are discovered, then the question is settled. Voigtel has rightly remarked that, in some moles—

"From an originally imperfect development of the ovum, or an injury to the fœtus at its first formation, it appears either as a shapeless mass, or *the fœtus itself is completely destroyed,* and *only its membranes and the placenta* continue to grow for a time and get thickened and fleshy, or filled with fluid only, or form membranous, fibrous masses, or hydatids, or assume other unnatural appearances." 22.

A minute and very delicate examination of the discharged mass, under the surface of water, is the only method by which we can hope to unravel and shew the anatomy of its structure; and Dr. Montgomery, from repeated experience, assures us, that if we will but take the necessary trouble and time, we shall generally be enabled to decide upon its true nature. The mole is to be first very carefully cleansed from all adhering blood by repeated ablutions, and the dissection is to be performed with delicate, blunt instruments. His observations are so interesting, that we shall give them in his own words.

" If in the progress of such an investigation we discover a fœtus, or even a part of one, it would of course be decisive; but this may not be the case, and yet we may recognise all the other component parts of the ovum presenting several structures which are never produced by disease.* First, we may have the decidua covering either partially or completely the substance under examination, distinguished by its soft, rich, pulpy appearance and strong red colour, its external or uterine surface rough and unequal, and, when well freed from the coagulated blood, exhibiting numerous small round foramina,† capable of admitting a pin: its internal surface is smooth, and exhibits little or no appearance of these openings. This coat may be found attached to the ovum, or entirely torn away and separated from it during its expulsion; but in either case these characters mark the true decidua, and are not found in the products of disease. Within this outer coat another is found immediately investing the membranes of the ovum, the outer surface of which is smooth, and its inner completely filamentous, receiving the beautiful arborescent villi which cover and shoot from the surface of the chorion, forming the bond of union between it and this inner decidua. The discovery of these arborescent villi or capillaries is proof positive of the nature of the product, as they are never found presenting like characters, except upon the chorion or uterine surface of the placenta." 21.

This description, it will be understood, applies in a particular manner to what may be called the " embryotic moles," those, namely, which are produced by a blighted or otherwise imperfect ovum; and if the accuracy of its details, especially as regards the decidua membrane, may be altogether depended upon, a ready and very convenient method of distinguishing the mole of conception from the casual and independent mole is furnished. But the very circumstance of the decidua being, in many cases, retained, and the surface of the mass being then quite smooth and glistening, and devoid of the shagginess which is characteristic of this membrane, is a sufficient proof that we cannot with safety rely on one feature alone. A case which very lately came under our notice, is worthy of notice on several accounts.

A lady, the mother of six children, was seized with the symptoms of threatened abortion, in consequence of the jolting of a carriage on an uneven road. By rest, and the use of refrigerant medicines, they were checked for a time; but after the lapse of two weeks they returned, and as the patient's health began to suffer much, and the quantity of blood which, at repeated discharges, had been lost, precluded the hope of ultimately preventing the miscarriage, it was determined, in consultation with one of the most eminent accoucheurs in London, rather to accelerate than to endeavour to retard it. The os uteri was open enough to admit the point of the finger, and a thin, mucous discharge dribbled from the orifice. An elastic gum bougie was, therefore, introduced, and, being pushed up into the cavity of

---

* " See a case related by Mr. Lemon in the Edinburgh Medical and Surgical Journal, vol. xi. p. 96. The writer has in his museum more than one specimen illustrative of this absence of the fœtus where the other parts of the ovum exist."

† See Hunter's plates of the gravid uterus, xxix. fig. 11, and also plates xxviii., xxx., xxxiii., xxxiv.

the womb, was permitted to remain there for twenty hours; on the following day a larger one was substituted, and a brisk purgative administered. During the operation of the medicine, after some sharp pains felt in the region of the womb, the patient was sensible of something having come away from her. It was examined and found to consist of four sanguineous lumps, which had apparently been joined together and formed a globular mass, but which had been torn during the expulsion. These lumps were about an inch thick, and had the appearance of densely coagulated blood, from which, in some parts, the colouring matter had been removed, so as to leave only the fibrinous portion. The outer or uterine surfaces were smooth and uniform; the inner presented a net-work of fibrinous fasciculi, not unlike that of the columnæ carneæ in the ventricles of the heart.

On introducing the finger into the vagina, the mouth of the womb was found hard and jagged, and not large enough to permit it to be passed into its cavity; so that no part of a fœtus could be felt. The patient being now comfortable, enjoyed some hours of refreshing sleep, and upon visiting her next morning, we found her better in every respect. The impression upon our minds was, that the womb had emptied itself of all its contents, and that in short, there had been a false conception. Twenty-four hours afterwards, a fœtus, of about three and a half months, with the chord and placenta, adhering and quite entire, but with no distinct membranes, was expelled with very little pain.

We have adduced this case to prove that we must not always expect to find the villous and shaggy surfaces in even indubitable products of conception; and moreover that these products may resemble most closely in many particulars, some of the other substances which have been denominated moles, and which many authors assure us, may be formed in females who never have had any sexual intercourse.

A useful lesson for the exercise of caution in giving our professional opinion, may likewise be drawn from the preceding history. Dr. Montgomery mentions a case in which a similar substance was expelled immediately after the discharge of a healthy ovum, containing a well-formed fœtus of four months.

"The substance had the external characters usually considered as those of a mole, and was of the form and size of a large orange. When opened, no trace of a fœtus could be discovered, but there was a small remnant of an umbilical cord, which was ragged at its unattached extremity: the fleshy envelope varied in thickness from an eighth to half an inch, the thickest part being that where the placenta was situated, the internal surface of which exhibited very remarkably the tubercular disease represented in Denman's ninth plate." 22.

On the whole, from an attentive examination of the sentiments of the most eminent obstetrical writers, we are disposed to agree with the conclusion to which our author has arrived; viz. that all moles which exhibit traces of the component structures of the ovum, such as are detailed in the description which we have extracted from Voigtel, are the results of impregnation, and are never discovered in the virgin womb. While however we make this admission, our earnest advice is, to exercise the greatest circumspection, to enquire diligently into all the accompanying circumstances of the case in question, and to avoid any rash and unpremeditated decision.

The subject is not yet "hors de combat." A similar remark is applicable to another uterine formation, that of hydatids; Denman, Gardien, and Sir C. Clarke admitting their accidental and independent development; while Baudelocque, Voigtel, and the French writers of the present day, regard them as invariably the products of conception.

### On the Corpora Lutea.

If we examine the ovaries of a woman, within a few days after impregnation, we shall find that one of these organs exhibits traces of the recent escape of the germ; it is larger and more vascular than the other, and is also fuller and softer to the touch; but this increase of size is not uniform over the whole surface; one point projects more than the rest of the gland, and if this part be carefully observed, a small slit or torn orifice may be seen there. Sir E. Home describes it in a woman who died, eight days it is supposed, after impregnation, as follows :—

"The right ovarium had a small torn orifice upon the most prominent part of its external surface. We slit it open in a longitudinal direction, in a line close to the edge of this orifice; the orifice was found to lead to a cavity filled up with coagulated blood, and surrounded by a yellowish organized substance." 32.

This projecting part of the ovarium always indicates the spot whence the vivified ovum has escaped; and if the examination is made soon after that event, there is seldom any difficulty of recognizing it; the appearances however become less and less distinct, according to the length of the interval which has elapsed since impregnation, and finally they cease altogether, with the exception of the cicatrix of the wound, which continues longer than the rest.

We can trace the series of changes more easily and satisfactorily in some of the lower animals; in the cow and sheep for example, the swollen part of the ovary projects we are told by Dr. M. as a parasitic tumor hanging from it; and in the common sow "the ovaries after conception appear literally like branches of round berries, from the great prominence of the numerous corpora lutea." The next step in the investigation, is to ascertain the internal structure and appearances of the part; and as it is of great importance that strictly accurate notions should be had on this subject, we cannot do better than extract the following description, which is given in Dr. Montgomery's Memoir, of a true corpus luteum.

"In *form* and *size* it is almost always an oval, with its longer axis varying from four to five eighths of an inch, and the shorter from three to four eighths; its thickness is generally less than its breadth.

*Its texture* is obviously and strikingly glandular, resembling a section of the human kidney; or, as some one has said, it is like a miniature of the particular sections of the brain called by anatomists *centrum ovale*. William Hunter describes is as "tender and friable, like glandular flesh."

*It is very vascular,* small vessels being very frequently visible without any preparation; but if fine-coloured injections have been previously thrown into one of the branches of the spermatic arteries going to the ovary, the vessels of the corpus luteum will be filled with the colouring matter, and are to be seen very distinctly running from its circumference towards its centre.

*Its colour* is, as its name implies, a dull yellow, very similar to that of the buffy coat of the blood; exhibiting generally, when recently exposed, a slightly reddish tinge, '*ex flavo rubens.*' Haller." 32.

We have seen that the cavity of the corpus luteum was found by Sir E. Home filled with coagulated blood, in a woman, who died eight days after conception; this blood becomes gradually absorbed; the little cavity becomes lined and surrounded with a tough white membrane; its dimensions are contracted, so that in three, or four months, it is often not larger than a grain of wheat, and subsequently it is entirely obliterated; and in its place there remains only an inner or central white radiated cicatrix; this cicatrix forms an essential character, we are told by the author, distinguishing the mark of the corpus luteum from that of any other formation, which may be confounded with it: it is not however permanent.

"The exact period of its total disappearance we are unable to state; but we have found it distinctly visible so late as at the end of five months after delivery at the full time, but not beyond this period; and the corpus luteum of a preceding conception is never to be found along with that of a more recent, when gestation has arrived at its full term; but in cases of miscarriage repeated at short intervals, it may." 32.

The following abstract of three dissections, will illustrate the appearances of the corpora lutea, in their different stages. *Obs.* 1. A woman died of inflammation of the womb, a few days after delivery; the white central cicatrix was very distinct; and externally the ovary exhibited the superficial cicatrix, and the swelling, or projection of the part. *Obs.* 2. In a woman who died in five weeks after delivery at the full time, the corpus luteum was found to be diminished to one half of its original dimensions; its texture had become firmer and more consolidated, and the yellow colour was indistinct in numerous points, so that it was much paler throughout, than at an earlier period; the radiated central cicatrix was quite distinct: the external surface of the ovary was fuller and more prominent over this part, and the cicatrix of the superficial fissure was well marked. Although this woman had borne six children, there was only one cicatrix observable on each ovary.

*Obs.* 3. In a woman, who died twelve weeks after delivery, the external swelling on the ovary was greatly diminished, but it was still sufficiently obvious to indicate the exact situation of the corpus luteum; the superficial cicatrix was well seen; the corpus luteum itself had lost much of its colour; and what remained, became, on immersion into spirits, of a light grey shade; the texture of its substance was more condensed, and resembled that of a cut apple; its dimensions, especially in breadth, were reduced to about one third, or rather less; but the central radiated cicatrix was still distinctly observable.

In a young woman who died five months after the delivery of her first child, the ovary retained very little of its increased size or altered form; the prominence was hardly to be recognized; but the external cicatrix was perfectly obvious. When opened the corpus luteum exhibited its peculiar colour, only in one very small spot, rather larger than a mustard seed, within which was observed the central radiated cicatrix; the yellow colour completely disappeared when the ovary was immersed in proof spirit, which does not happen with a corpus luteum examined during gestation, or about the period of delivery.

It is quite an erroneous supposition that corpora lutea are permanent during life, and that we can predict from the appearance of the ovaries, the number of children which the woman has borne. Dr. M. has never been able to detect their existence when more than five or six months from the time of delivery have elapsed. Even the external cicatrix becomes gradually less distinct, and may be at length quite effaced, so that the ovaries of women who have borne many children, may present only one or two scars, and perhaps none at all, if the interval since the last gestation has been long.

It is to be remembered, that if the ovaria become diseased, and especially if they have ever been the seat of suppuration, scars very like to those left by the escape of ova, may be formed, and thus give rise to very serious mistakes. The conclusion which Dr. M. draws from his repeated observations and dissections of a great number of women, and a much larger number of brute animals, is that he "has never in any one instance seen the corpus luteum, having the characters as above described belonging to it, except in females who had previously been impregnated, and who had conceived; and that such a corpus luteum was never found in a virgin animal." And again he says, "we believe no one ever found a fœtus in utero without a corpus luteum in the ovary; and that the truth of Haller's corollary 'nullus unquam conceptus est absque corpore luteo,' remains undisputed." Such also were the sentiments of De Graaf, of Dr. Haighton, and of Mr. Cruickshank. Occasionally indeed a corpus luteum has been discovered without a fœtus, or the number of the corpora may exceed the number of the fœtuses which are developed at the time. The great master of physiology has noticed this seeming incongruity, in his Elementa—"si unquam absque fœtu, corpus luteum in ovario repertum est, quod est rarissimum, credibile est eum fœtum abortu perditum, aut alio modo destructum disparuisse." Having thus ably explained the characters of the genuine corpora lutea, our author proceeds to examine the opinions of those physiologists who have maintained, that they may be formed in the ovaries of virgin females, and in no part of his memoir has he been more felicitous and conclusive, than in his refutation of those doctrines. He most satisfactorily shews that authors have upon this, as too often upon other subjects, which can only be determined by minute and repeated personal observations, idly followed each other's assertions, and have rarely hazarded their opinions upon their own authority. The testimony of the distinguished Blumenbach has been frequently referred to on this subject, and yet it is very remarkable, that in no one part of his dissertation does even he "speak as from personal observation or examination of the subject by himself, but confines himself to physiological reasonings grounded on the facts observed by others,* from the consideration of which he declares his *belief* † in one place, and his *suspicion* ‡ in another, that the fact may be so, but he nowhere asserts that he saw an instance of it; and he adds that all the cases his reading furnished him with, happened

* "'Corpora lutea in innuptis observarunt *auctores*.' Op. cit. p. 113."

† "'Et ita corpora lutea in virgineo corpore oriri *confido*.'"

‡ "'Non absimilem originem *suspicor*.' Op. cit. p. 113."

in Italian girls, whose climate he appears to suspect might have something to do with the matter."

Meckel, too, who has been adduced in confirmation of the same views, nowhere distinctly asserts that a true corpus luteum is ever seen in the virgin ovary; his words are—

"The influence of the male semen is the ordinary and regular cause of this change, which, however, *it appears,* may be effected under the influence of other stimuli, *perhaps* by the imagination or unnatural enjoyments."

And again—

"In truth, many of these rare cases, in which corpora lutea have been found in unmarried women, and in girls having the physical marks of virginity, allow the belief that the formation of these bodies *had been preceded by sexual intercourse and fecundation.*" 35.

Now surely these hesitating, and at best only conjectural opinions, are far outweighed by the decisive evidence, drawn from a multitude of experiments performed on purpose, by the authors to whom we formerly alluded. No words can be stronger than those employed by Dr. Haighton in a paper read before the Royal Society of London.

"I decline trespassing on your patience, and therefore lay before you only the conclusion; which is, that in the great variety of experiments on brute animals which my physiological inquiries have led me to conduct, as well as in the extensive opportunities I have had of observing the ovaries in the human subject, I have never seen a recently formed corpus luteum unattended with some circumstance or other connecting it very evidently with impregnation." 36.

With the truth of these remarks, the most eminent anatomists of the present day coincided upon the trial at Liverpool, in 1808, of Mr. Angus, for the supposed murder of Miss Burris.

"It was not until after the trial that the ovaria were examined. They were then divided in the presence of a number of physicians, and a corpus luteum distinctly perceived in one of them. Mr. Hay took the uterus and its appendages to London, and shewed it to the most eminent practitioners there. He received certificates from Drs. Denman and Haighton, Messrs. Henry Cline, Charles M. Clarke, Astley Cooper, and Abernethy, all stating that it exhibited appearances that could alone be explained on the idea of an advanced state of pregnancy. *And it appears to have been universally allowed, that the discovery of the corpus luteum proved the fact beyond a doubt.*" 37.

Respectable writers have frequently described appearances in virgin ovaries, which most inaccurately have been mistaken for true corpora lutea; and, indeed, every one who is in the habit of examining dead bodies must have upon many occasions found yellow spots in these organs, quite unconnected with any previous impregnation; but these different appearances may be distinguished by the accurate observer.

"We think that those who have supposed or asserted that they may exist without impregnation, and of course be found in the virgin ovary, have been led into the error by confounding appearances and structures essentially different, and in fact having only one character in common, which is their colour, altogether forgetting that 'every yellow substance in the ovary is not a corpus luteum.'* It is allowed by those writers that 'the corpora lutea of virgins may in general be distinguished by their smaller size, and by the less extensive vas-

* "*Meckel,* supra citat."

cularity of the contiguous parts of the ovarium.'* Now we have seen several of these virgin corpora lutea, as they are unhappily called, and have preserved several specimens of them, and according to our experience they differ from those of impregnation in all the following particulars:—1. there is no prominence or enlargement of the ovary over them; 2. the external cicatrix is wanting; 3. there are often several of them in both ovaries, especially in patients who have died of tubercular diseases; 4. they are not vascular, and cannot be injected; 5. their texture is sometimes so infirm, that they seem to consist merely of the remains of a coagulum, and at others appear fibro-cellular and resembling that of the internal structure of the ovary, but in no instance did we ever see them presenting the soft, rich, and regularly glandular appearance which Hunter meant to express when he described them as 'tender and friable like glandular flesh;'† 6. they have neither the central cavity, nor the radiated cicatrix which results from its closure." 37.

Our reasons for having selected the subject of the corpora lutea for illustration, in preference to the other post-mortem signs of impregnation, must be now abundantly evident. Hitherto there has been nothing like certainty, and no congruity of sentiments, upon the true nature and the true origin of these formations. Upon a trial which took place some years ago in Edinburgh, the most opposite and inconsistent evidence was given by the medical men who were examined as witnesses. Four students had exhumed the body of an elderly female, who was unmarried and a virgin; and when they were apprehended, the corpse was found to be so disfigured, that no accurate identification could be made. It was alleged in the defence of the prisoners, who denied the charge, that a genuine corpus luteum had been found in one of the ovaries; and a number of surgeons were summoned to pronounce upon this appearance, and, at the same time, to determine whether it should be admitted as a proof of the person having been ever impregnated. One half of them maintained the affirmative of both these questions, and the other half were of the opposite opinion, so that no safe deductions could possibly be drawn from the medical evidence. The body was afterwards identified, by a dentist producing a cast which he had taken of the gums.

Several of the other topics treated of in this Essay are equally interesting and instructive, especially those which relate to the occasional occurrence not only of conception, but even of delivery, without the consciousness of the female, while asleep, or during the stupor of an hysterical paroxysm. Some may, perhaps, be inclined to smile with incredulity at the bare mention of such possibilities; let them smile on, if it pleases them, but let them cease their incredulity—or rather, we should term it, their ignorant presumption. Capuron, Fodéré, Marc, Dr. Gooch, Mr. Cusack, and many others, have met with cases which fully prove the first of these positions. The language of the first of these authors is very explicit.

"'It is a fact (says Capuron) which experience has more than once confirmed, that a woman may become with child while in a state of hysteria, under the influence of narcotics, during asphyxia, drunkenness, or *deep sleep*, and consequently without being conscious of it, or sharing the enjoyment of the man who dishonours her;' and in proof he mentions having attended a young woman who was got with child while totally unconscious, being buried in a deep sleep pro-

* Mr. Stanley and Dr. Blundell.
† Description of Gravid Uterus, p. 14.

duced by punch given her by her paramour.* She became aware of her condition for the first time when she felt the sensation of motion in the fourth month." 28.

Equally unimpeachable are the authorities which may be adduced, to prove that delivery has actually taken place, without any consciousness of the fact.

"In the London Practice of Midwifery,† a work generally ascribed to a late very distinguished practitioner, we find the following account. 'A lady of great respectability, the wife of a peer of the realm, was actually delivered once in her sleep: she immediately awaked her husband, being a little alarmed at finding one more in bed than was before.'" 44.

The possibility of these occurrences ought ever to be kept in mind by the medical jurist and the friend of humanity; for we have seen that a female may not only incur all the moral turpitude of dissoluteness, but even be exposed to the aggravated charge of infanticide; and yet be unconscious of the act of infamy on the one hand, and of her delivery and of the death of the child, on the other.

Those who are anxious for particulars should consult the original essay; they will be amply rewarded by a diligent perusal of it. It contains a vast quantity of highly useful professional information, and, on the whole, is one of the most able contributions to the excellent Cyclopædia of Practical Medicine.

* See Méd. Lég. relat. aux Accouchemens, pp. 57, 84.

† Fifth Edition, p. 87. See also Barlow's Essays on Surgery and Midwifery, p. 182.

